# Pluripotency and X chromosome dynamics revealed in pig pre-gastrulating embryos by single cell analysis

**DOI:** 10.1038/s41467-019-08387-8

**Published:** 2019-01-30

**Authors:** Priscila Ramos-Ibeas, Fei Sang, Qifan Zhu, Walfred W. C. Tang, Sarah Withey, Doris Klisch, Liam Wood, Matt Loose, M. Azim Surani, Ramiro Alberio

**Affiliations:** 1 School of Biosciences, University of Nottingham, Sutton Bonington Campus, Nottingham, LE12 5RD UK; 2 School of Life Sciences, University of Nottingham, Nottingham, NG7 2RD UK; 3 Wellcome Trust/Cancer Research UK Gurdon Institute, University of Cambridge, Tennis Court Road, Cambridge, CB2 1QN UK; 4 Department of Physiology, Development and Neuroscience, University of Cambridge, Downing Street, Cambridge, CB2 3DY UK; 5 Wellcome Trust Medical Research Council Stem Cell Institute, University of Cambridge, Tennis Court Road, Cambridge, CB2 1QR UK; 6 Present Address: Animal Reproduction Department, National Institute for Agricultural and Food Research and Technology, 28040 Madrid, Spain; 7 Present Address: Stem Cell Engineering Group, Australian Institute for Bioengineering and Nanotechnology, University of Queensland, Building 75, St Lucia, QLD 4072 Australia

## Abstract

High-resolution molecular programmes delineating the cellular foundations of mammalian embryogenesis have emerged recently. Similar analysis of human embryos is limited to pre-implantation stages, since early post-implantation embryos are largely inaccessible. Notwithstanding, we previously suggested conserved principles of pig and human early development. For further insight on pluripotent states and lineage delineation, we analysed pig embryos at single cell resolution. Here we show progressive segregation of inner cell mass and trophectoderm in early blastocysts, and of epiblast and hypoblast in late blastocysts. We show that following an emergent short naive pluripotent signature in early embryos, there is a protracted appearance of a primed signature in advanced embryonic stages. Dosage compensation with respect to the X-chromosome in females is attained via X-inactivation in late epiblasts. Detailed human-pig comparison is a basis towards comprehending early human development and a foundation for further studies of human pluripotent stem cell differentiation in pig interspecies chimeras.

## Abstract (web summary)

Lineage segregation from conception to gastrulation has been mapped at the single cell level in mouse, human and monkey. Here, the authors provide a comprehensive analysis of porcine preimplantation development using single cell RNA-seq; mapping metabolic changes, X chromosome inactivation and signalling pathways.

## Introduction

Pre-gastrulation embryo development shows broad similarities between mammals, although species-specific differences in early lineage segregation, the establishment of pluripotency, and X-chromosome inactivation have been reported^1–3^. Mouse embryos, which are widely used as a model for mammals, transit rapidly through this early development phase (E0-E5.5) that culminates with the formation of the characteristic cup-shaped post-implantation epiblast. In larger mammals, including humans, non-human primates (NHP) and pigs, there is a protracted developmental period (~10–12 days) that ends with the formation of a flat bilaminar embryonic disc. Since early post-implantation human embryos are largely inaccessible, and currently can only be studied with novel in vitro systems^4,5^, we are beginning to investigate relatively more accessible pig embryos. Notably both human and pig embryos evidently form a flat embryonic disc before the onset of gastrulation^6^. Thus, the pig embryo can broaden our understanding of the pre-gastrulation development of large mammals with protracted development.

Segregation of trophectoderm (TE) and hypoblast, and the emergence of pluripotency are well established in mice, but require detailed studies in other mammals at the resolution of single cells, as recently reported for *Cynomolgus* monkeys^2^. Potential discrepancies in lineage segregation have however emerged in reports between monkey and human, attributed in part to embryo staging differences^7^. Further studies, including those in other large mammalian species, are therefore highly desirable.

In mouse embryos a distinct transcriptional signature of pluripotency in the inner cell mass (ICM) undergoes changes as the epiblast (EPI) matures and develops further marking a transition through pluripotency before gastrulation^8^. These transitory stages can be recapitulated in vitro in naive pluripotent stem cells (PSCs), which resemble pre-implantation epiblast cells, and primed PSCs resembling the post-implantation mouse epiblast^9^. Establishment of similar cell lines from non-rodent mammalian species, including humans, has been challenging, suggesting possible biological differences^10^. Indeed, spatiotemporal differences in the expression of core pluripotency genes *NANOG, OCT4* (*POU5F1*) and *SOX2* have been noted, while the expression of *Klf2*, *Prdm14* and *Bmp4*, observed in mouse embryonic naive cells, are apparently not detectable in human embryos^10,11^. By contrast, *KLF17* is expressed in the human but not mouse ICM^10–12^. Also, while members of the Jak-Stat3 and WNT signalling pathways are detected in the early mouse ICM^13^, many TGFβ signalling components are found in marmoset, human and pig ICM^11–14^, indicating that the emergence and establishment of pluripotency in mammals is controlled by different signalling pathways and gene networks. Differences in the mechanisms of X-linked gene dosage compensation in female embryos are also evident^3^. The gene dosage compensation with respect to the X chromosomes in female embryos occurs in pre-gastrulation epiblasts in mouse and rabbits^3,8,15^. Notably, human post-implantation and pig pre-gastrulation epiblasts have not been studied^12,15^.

Here we report lineage segregation, the establishment of pluripotency, and X-chromosome inactivation during the entire peri-gastrulation period in the pig embryo using single-cell RNA-seq (scRNA-seq). This comprehensive analysis provides new understanding of the developmental trajectories of early embryonic cells in the pig, which shares similarities with early human development, and other mammals with similar embryology.

## Results

### Progressive lineage segregation in pig embryos

**Fig. 1:**
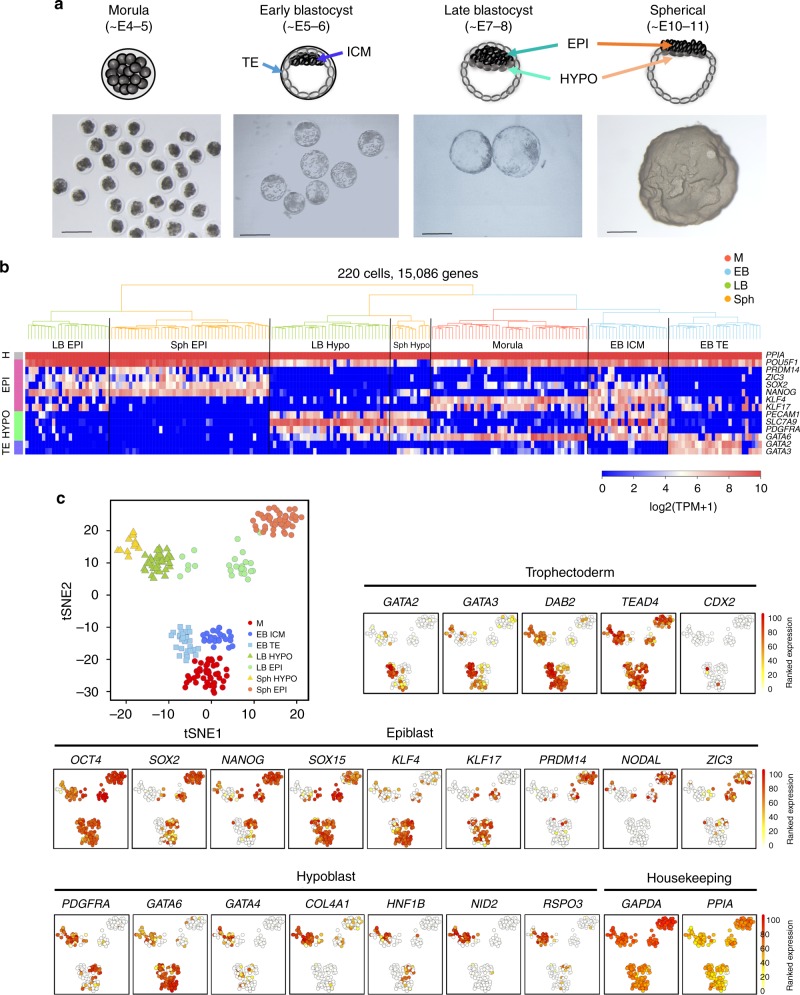
Lineage segregation in pig pre-implantation embryos. **a** Pig pre-implantation embryos collected for scRNA-Seq. **b** Unsupervised hierarchical clustering (UHC) with all expressed genes (15,086 genes), with a heat map of expression levels of lineage-specific markers. Colours in dendrogram indicate developmental stage. **c** t-SNE plot of all cells, indicated by colours and shapes for different embryonic days and lineages. Lineage-specific genes are shown in t-SNE plots; a gradient from white to red indicates low to high expression. *n* = 220 cells. E: embryonic day, M: morula, EB: early blastocyst, LB: late blastocyst, Sph: spherical embryo, EPI: epiblast, HYPO: hypoblast, ICM: inner cell mass, TE: trophectoderm. Scale bar: 300 µm

First, we set out to generate a single-cell transcriptome profile of early in vivo pig embryo development, from four pre-implantation stages: morula (M; embryonic day (E) ~4–5), early blastocyst (EB, ~E5–6), late blastocyst (LB, ~E7–8), and spherical embryo (Sph, ~E10–11)^16^ (Fig. 1a), and obtained 220 single-cell transcriptomes from 28 embryos (Table 1, Source data file). Unsupervised hierarchical clustering (UHC) (15,086 genes) grouped the cells according to their developmental stage and specific lineages based on known markers (Fig. 1b).

**Table 1:** Samples of embryos collected

Stage	No. of embryos	No. of cells collected	No. of cells sequenced	Samples retained after quality control
Morula (~E4–5)	8	91	58	47
Early blastocyst (~E5–6)	10	158	57	52
Late blastocyst (~E7–8)	6	103	70	61
Spherical embryo (~E10–11)	4	113	65	60
Total	28	465	250	220

Dimensionality reduction provided a clear visualisation of lineage segregation during development (Fig. 1c). The morula group showed expression of *OCT4* (*POU5F1)*, *SOX2*, and *KLF4*, but not *NANOG*, while early blastocyst (EB) cells segregated into two lineages: ICM cells expressing *NANOG* and *SOX2*, and TE cells with *GATA2*, *GATA3* and *DAB2*. Expression of *CDX2* was seen in a few TE cells at this early stage^14^, but *OCT4* expression was seen in all cells, consistent with observations in human and monkey blastocysts^2,17^. There was evident expression of pluripotency genes; *SOX15*, *KLF4* and *KLF17* in the ICM and EPI, as in human epiblast cells. Expression of some of these genes was also seen in pig TE and hypoblast (HYPO).

**Fig. 2:**
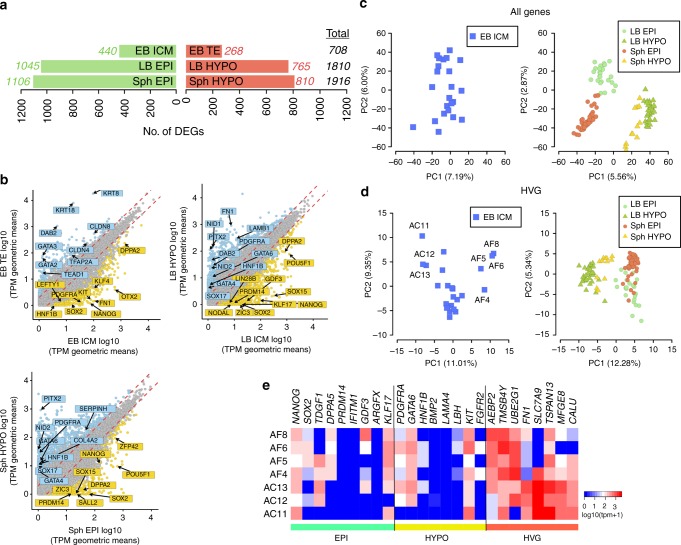
Differential gene expression in cells of the early pig embryo. **a** Numbers of differentially expressed genes (DEGs) during lineage segregation in pairwise comparisons for each stage. Red and green bars indicate upregulated and downregulated genes, respectively. **b** Scatter-plot comparisons of the averaged gene expression levels between different lineages (>1 fold change, flanking diagonal lines; Yellow: upregulated, blue: downregulated; log_10_ (TPM geometric means), key genes are annotated). **c** PCAs of EB ICM (*n* = 24 cells) and LB and Sph EPI and HYPO of all expressed genes (*n* = 121 cells). **d** PCA of EB ICM (*n* = 24 cells) and LB and Sph EPI and HYPO cells by highly variable genes (HVG) (n = 121 cells). **e** Heat map of expression levels of epiblast- and hypoblast-specific markers and HVG in seven selected EB ICM samples from **c** and **d**. M: morula, EB: early blastocyst, LB: late blastocyst, Sph: spherical embryo, EPI: epiblast, HYPO: hypoblast, ICM: inner cell mass, TE: trophectoderm

We identified 708 differentially expressed genes (DEG) between ICM and TE (Fig. 2a and Source data file). While *GATA2* and *GATA3* were the two top-ranked genes in TE of early blastocysts, other TE markers reported in mouse and human such as *ANXA6* and *TEAD1* were identified for the first time in the pig. Notably, we found upregulation of both HYPO and EPI markers in the ICM (Fig. 2b). Further interrogation of ICM cells by principal component analysis (PCA) of all genes and highly variable genes (Fig. 2c and d, respectively) did not separate the cells into discrete populations. Analysis of highly variable genes (HVGs) in a subset of cells separated along PC1 did not show a distinct EPI or HYPO expression signature based on high-confidence markers^7^ (Fig. 2e). Mutually exclusive segregation of EPI and HYPO became evident first in cells of LB and Sph embryos (TE was excluded from these stages) (Fig. 2c, d). Expression of *SOX2*, *NANOG*, *PRDM14* and *NODAL* was observed in EPI, whereas expression of *PDGFRα*, *GATA4*, *GATA6*, *COL4A1*, *NID2* and *HNF1β* was detected in HYPO (Fig. 1c). Comparison between EPI and HYPO in LB and Sph identified 1810 and 1916 DEGs, respectively. Known EPI genes upregulated in both stages included *SOX15*, *ZIC3*, *FGF19*, *SALL2* and the HYPO genes *PITX2*, *PECAM1, DAB2* and *FN1* (Fig. 2a, b, and Source data file). These results show that TE and ICM in the pig embryo segregate in the early blastocyst, whereas at this stage, HYPO and EPI genes are co-expressed in the ICM; these cells resolve into discrete cell lineages in late blastocysts.

### Signalling pathways controlling lineage segregation

Gene ontology (GO) enrichment and Kyoto Encyclopaedia of Genes and Genomes (KEGG) pathway analyses indicated that PI3K-Akt and Jak-Stat signalling pathways were over-represented in ICM and TE of early blastocysts (EB), but the WNT signalling pathway was enriched only in ICM cells. In later stages, PI3K-Akt was over-represented in HYPO and MAPK signalling in EPI. Components of the TGFβ pathways were expressed in both EPI and HYPO (Table 2 and Source data file).

**Table 2:** Gene ontology terms and KEGG pathways determined by pairwise comparisons of embryonic lineages

Lineage	GO terms	KEGG pathways
EB ICM vs. EB TE		
EB ICM	Endodermal cell fate specification (7.07e−04) IL-6-mediated signalling pathway (1.05e−03) Positive regulation of canonical Wnt sig. path. (1.28e−03) Positive regulation of JAK-STAT cascade (2.37e−03) Stem cell population maintenance (4.00e−03)	Signalling pathways regulating pluripotency of stem cells (2.43e−06) Calcium signalling pathway (8.48e−06) Cell adhesion molecules (CAMs) (3.53e−04) PI3K-Akt signalling pathway (5.57e−04)
EB TE	Regulation of establishment of cell polarity (5.00e−04) Positive regulation of cell migration (7.13e−04) Cell proliferation (2.01e−03) Positive regulation of cell proliferation (3.71e−03) Positive regulation of JNK cascade (4.11e−03)	PI3K-Akt signalling pathway (1.23e−02) Phagosome (1.31e−02) Jak-STAT signalling pathway (3.17e−02) Lysosome (4.56e−02) Cell cycle (8.03e−02)
LB EPI vs. LB HYPO		
LB EPI	Negative regulation of transcription from RNA polymerase II promoter (3.92e−05) Histone H4-K5 and H4-K8 acetylation (1.69e−03) Regulation of DNA methylation (1.10e−02) C-5 methylation of cytosine (2.50e−02) Chromatin silencing (4.13e−02)	MAPK signalling pathway (5.21e−03) Neuroactive ligand-receptor interaction (2.55e−02) Signalling pathways regulating pluripotency of stem cells (3.20e−02) ErbB signalling pathway (3.66e−02) Adherens junction (3.75e−02)
LB HYPO	Transmembrane transport (4.11e−05) Response to glucose (1.35e−04) Cholesterol efflux (1.63e−04) Endodermal cell differentiation (1.81e−03) Triglyceride homoeostasis (2.12e−03)	PI3K-Akt signalling pathway (4.87e−06) ECM–receptor interaction (6.09e−06) Cell adhesion molecules (CAMs) (5.98e−05) Focal adhesion (2.16e−04) PPAR signalling pathway (3.87e−04)
Sph EPI vs. Sph HYPO		
Sph EPI	Axon guidance (1.03e−04) Regulation of neuron projection development (1.99e−04) Glycogen catabolic process (3.50e−03) Embryo implantation (7.88e−03) Regulation of stem cell division (1.17e−02) Stem cell population maintenance (1.25e−02)	PPAR signalling pathway (3.72e−22) HIF-1 signalling pathway (1.54e−11) Tight junction (3.69e−07) Signalling pathways regulating pluripotency of stem cells (6.67e−07)
Sph HYPO	Endodermal cell differentiation (8.04e−05) Endoderm development (1.74e−04) Lipid transport (2.54e−04) Response to glucose (2.84e−04) Cell fate commitment (5.31e−04)	ECM–receptor interaction (1.20e−04) TGF-beta signalling pathway (1.77e−04) Cytokine–cytokine receptor interaction (1.18e−03) PPAR signalling pathway (2.81e−03) Cell adhesion molecules (CAMs) (3.93e−03)

Significance values in brackets

*M* morula, *EB* early blastocyst, *LB* late blastocyst, *Sph* spherical embryo, *ICM* inner cell mass, *TE* trophectoderm, *EPI* epiblast

**Fig. 3:**
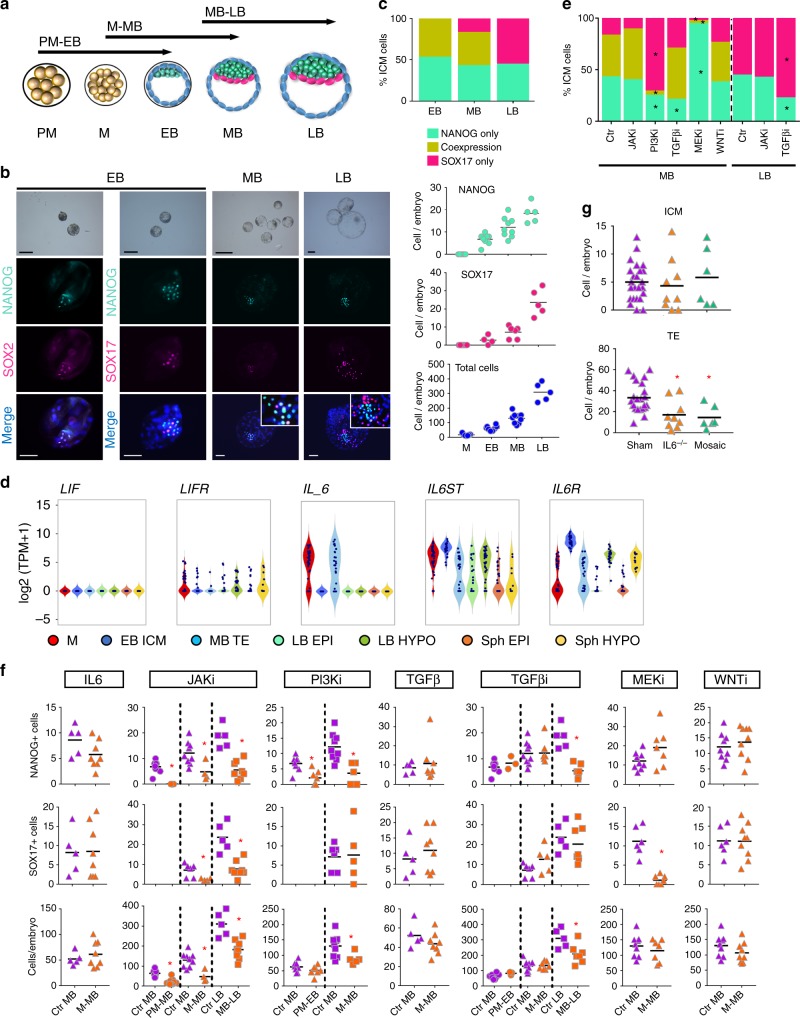
Signalling pathways involved in segregation of lineages. **a** Experimental design of embryo treatments. **b** Bright field and IF staining for indicated markers; embryos were counterstained with DAPI (merge). Scatter dot plots of NANOG-, SOX17-positive cells and total cell numbers (black bar indicates mean) of control pig morula (M, *n* = 7), early blastocysts (EB, *n* = 8), mid-blastocysts (MB, *n* = 9) and late blastocysts (LB, *n* = 5). **c** Bar charts indicating percentage of ICM cells expressing indicated markers in control embryos. **d** Gene expression of *LIF/IL6* and its cognate receptors in pig embryos. **e** Bar charts indicating percentage of ICM cells expressing indicated markers after different treatments. **f** Scatter plots show proportion of cells stained for the indicated markers in control embryos (EB *n* = 8, MB *n* = 9, LB *n* = 5) and embryos treated with JAKi: 10 µM AZD1480 (EB *n* = 11, MB *n* = 4, LB *n* = 8), PI3Ki: 10 µM LY294002 (EB *n* = 7, MB *n* = 5), TGFβi: 20 µM SB431542 (EB *n* = 3, MB *n* = 7, LB *n* = 7), MEKi: 10 µM PD0325901 (MB *n* = 7), WNTi: 3 µM IWP2 (MB *n* = 9), IL6: 10 ng ml^−1^ (MB *n* = 8) and TGFβ: 10 ng ml^−1^ (MB *n* = 8). **g** Scatter plots show the number of cells stained in Sham controls (*n* = 24), homozygous KO (*IL6*^−/−^) (*n* = 9) and mosaic embryos (*n* = 6) after CRISPR/Cas9 gene editing. ICM cells were determined by counting SOX2 positive cells and TE cells were calculated by subtracting SOX2 cells from the total cell count. Ctr: control, PM: pre-morula. For **c**, **e**: *p ≤ 0.05, two-way ANOVA. For **f**, **g**: *p ≤ 0.05, Mann–Whitney test. Source data are provided as a Source Data file. Scale bars: 50 µm

For elucidating functional roles of these signalling pathways during lineage segregation, we cultured ex vivo pig embryos at different stages based on the gene expression profile of the signalling pathways under investigation (Fig. 3a). We used selective inhibitors and determined the impact on lineage allocation by assessing the number of NANOG and SOX17 cells using immunofluorescence (IF). In controls, NANOG was absent in morulae, but detectable in most ICM cells from EB, which were also positive for SOX2 (Fig. 3b, c). Expression of SOX17 was first observed in a subset of NANOG positive (NANOG+) cells (47.14%) in the ICM of EB, which became gradually restricted to a small group of cells in the ICM of mid-blastocysts (MB, ~E6–7, 16.78%). By the late blastocyst (LB) stage (~E7–8), two mutually exclusive groups of NANOG+ and SOX17+ cells were identified (Fig. 3b, c).

Having established the sequence of NANOG and SOX17 expression, we first looked at Jak-Stat signalling, since it was highly represented in all cells of the EB (Table 2). In mouse, ICM cells express LIF receptor (LIFR)^18^ and glycoprotein 130 (also known as IL6ST), which bind LIF secreted by the neighbouring TE^19^. In the pig, however, *LIF* expression was not detected (Fig. 3d). Instead, *IL6* was expressed in M and EB TE cells. Similarly, high expression of *IL6ST* and *IL6R* was detected in ICM and TE cells of EB, which decreased in LB and Sph EPI (Fig. 3d). The effect of this growth factor during embryo development was tested by IL-6 supplementation to ex vivo-derived morulae. IL-6 supplemented embryos showed a modest increase in total cell number, with the proportion of NANOG and SOX17 cells unaffected (Fig. 3f). To further understand the role of IL6 during blastocyst formation we disrupted the *IL6* gene using CRISPR/Cas9 gene editing in pig parthenogenetic embryos and cultured these until the blastocyst stage. *IL6* KO (homozygous and mosaic embryos) had significantly reduced numbers of TE cells compared to sham injected embryos, however the ICM of these embryos was unaffected (Fig. 3g; Supplementary Figure 2). We also blocked Jak-Stat signalling in ex vivo embryos using a specific inhibitor (AZD1480) and found that early blastocysts had a significantly reduced total cell number and no clear ICM (Fig. 3f). While a small number of scattered SOX2+ cells were observed, they were however not organised into an ICM, unlike in control embryos (Fig. 3b). Embryos treated until the MB and LB stages also had reduced total cell numbers, as a result of a reduction of both the TE and ICM (Fig. 3e, f).

We also looked at the role of the PI3K-Akt signalling pathway previously identified in mouse pre-implantation embryos^20^, since we found it enriched in pig EB KEGG terms. Embryos treated with the PI3K inhibitor LY294002 from PM to EB and from M to MB developed small blastocysts with reduced numbers of NANOG+ cells compared to controls (3 cells in LY294002 vs. 10 cells in controls) (Fig. 3e, f). The total cell number was also reduced by more than 35 cells per embryo (Fig. 3f).

Next, we investigated the Activin/TGFβ pathway, which was previously shown to be active during EPI development in human and pig^11,21,22^. Supplementation of TGFβ1 at the morula stage did not affect the total cell number nor the proportion of NANOG and SOX17-positive cells in these embryos (Fig. 3f). We next blocked the Activin/TGFβ pathway using SB431542 (20 or 40 µM) from PM to EB and M to MB and found no effect in embryo development. In contrast, embryos treated from MB to LB showed a significant reduction in NANOG+ cells, but the number of cells expressing SOX17 was unaffected (Fig. 3e, f). These results indicate that TGFβ promotes the expansion of the pluripotent epiblast in the pig embryo without affecting lineage segregation, consistent with the findings in human embryos^11^.

In human, pig and cattle, inhibition of FGF signalling with a MAPK/ERK kinase inhibitor (PD0325901; 0.4–1 µM) does not abolish the expression of hypoblast markers^23–25^, in contrast to mouse and rabbit embryos where it prevents hypoblast formation^26,27^. As our scRNA-Seq data show expression of MAPK pathway genes in LB EPI cells, we tested the effect of the MEK inhibitor PD0325901 at high concentration (10 µM), based on previous results with cattle blastocysts^28^. MEK inhibition from M to MB significantly reduced the number of HYPO cells resulting in <3 SOX17+ cells/embryo, with an apparent shift towards NANOG+ cells in the ICM (Fig. 3e, f). This indicates that MAPK inhibition restricts the expansion of the pig HYPO, but it does not prevent the activation of SOX17 in some cells.

Lastly, since regulation of the canonical WNT pathway was a significantly upregulated GO term in the ICM, we cultured pig embryos with the tested WNT inhibitor IWP2. No reduction in hypoblast segregation nor the total cell number was observed following WNT inhibition from M to MB (Fig. 3e, f); similar observations were reported for mouse embryos^13^.

### Emergence and progression of pluripotency in the pig embryo

**Fig. 4:**
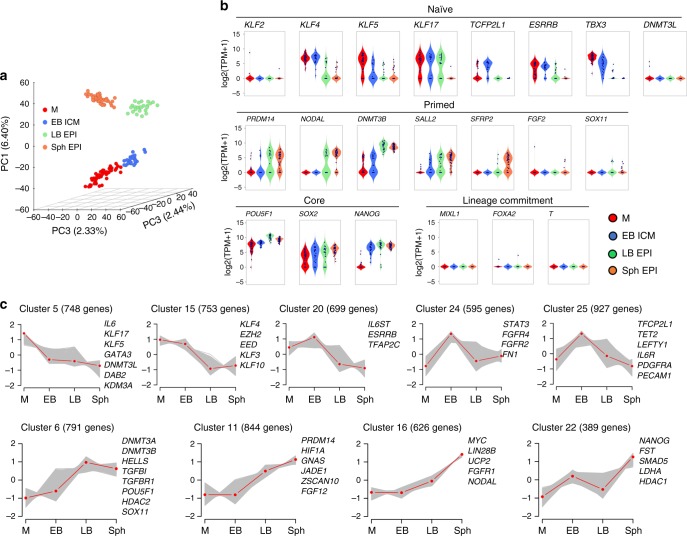
Progression of pluripotency in the pig embryo: **a** Principal component analysis (PCA) of the pluripotent lineages (*n* = 144 cells). **b** Violin plots of the expression of selected pluripotency and lineage specifier genes. **c** Self-organising maps (from a total of 25) showing key genes representative of naive and primed pluripotent cells

We next sought to determine how the emergent pluripotent cells (ICM) of EB compared to early (LB) and late EPI (Sph) (Table 3). In a three-dimensional PCA plot, cells grouped as two main clusters: M/ICM and LB/Sph EPI cells (Fig. 4a). We detected a biphasic profile of pluripotent gene expression, with high expression of naive pluripotency genes in M/ICM, and gradual downregulation of these markers in EPI cells (LB/Sph). Concomitantly with the decrease in naive markers, there was an upregulation of primed pluripotency genes in LB/Sph EPI (Fig. 4b). Essential differences in gene expression were noted in the pig compared to observations in the mouse^29^; while *OCT4* and *SOX2* expression were maintained along all pluripotent stages, expression of *NANOG* was first observed in the ICM and remained high in LB and Sph EPI. The naive pluripotency markers *KLF4*, *KLF5*, *KLF17*, *TFCP2L1*, *ESRRB* and *TBX3*, were detected in M and ICM and decreased, or even ceased in LB and Sph EPI. The exception was *PRDM14*, which followed the opposite trend. By contrast, primed pluripotency markers *NODAL*, *DNMT3B*, *SALL2* and *SFRP2* were upregulated in LB and Sph EPI. Continued expression of pluripotency markers and absence of lineage commitment gene expression (*MIXL1, FOXA2*, and *T*) in Sph EPI indicated a protracted exit from pluripotency (about 6 days) in the pig.

**Table 3:** Summary of female and male cells analysed

Stage	Cells		Embryos	
	Male	Female	Male	Female
M	32	15	5	3
EB ICM	18	6	4	6
EB TE	8	20		
LB EPI	13	12	3	3
LB HYPO	4	32		
Sph EPI	37	11	3	1
Sph HYPO	12	0		
Total	124	96	15	13

**Fig. 5:**
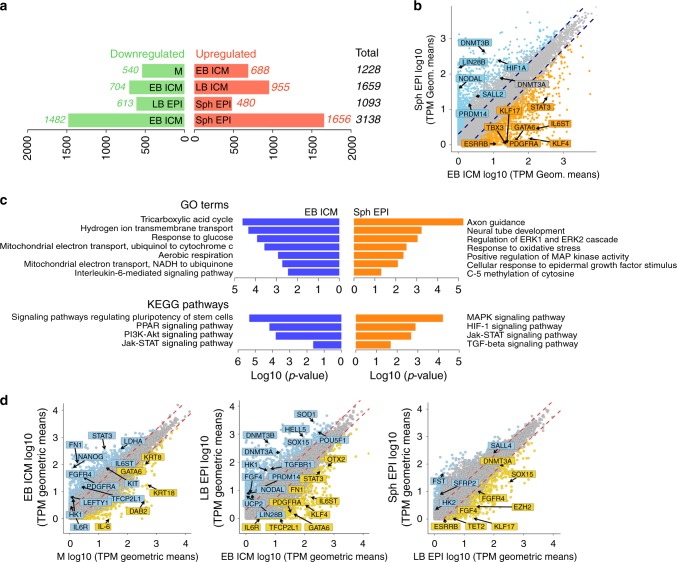
Gene expression changes during progession of pluripotency. **a** DEGs during the progression of pluripotency. Red and green bars indicate upregulated and downregulated genes, respectively, by pairwise comparisons as indicated. **b** Scatter-plot of the average gene expression levels between EB ICM vs. Sph EPI (>1 fold change flanking diagonal lines). Upregulated (orange) and downregulated (blue). Key genes are annotated. **c** Significant gene ontology terms and KEGG pathways in DEGs in the pairwise comparisons. **d** Scatter-plot comparisons of the averaged gene expression levels between M and EB ICM, EB ICM and LB EPI, and LB EPI and Sph EPI (>1 fold change, flanking diagonal lines; orange: upregulated, blue: downregulated; log_10_ (TPM geometric means))

We used K-means clustering to group genes with similar expression profiles (Fig. 4c). Genes highly expressed in morulae and ICM cells (cluster 5, 15, 20, 24, 25) include naive pluripotency markers, members of the Jak-Stat pathway, *TET2*, and components of the Polycomb Repressive Complex 2 (PRC2) *EZH2* and *EED*. Genes upregulated in LB and Sph EPI (cluster 6, 11, 16, 22) include primed pluripotency markers, DNA methyltransferases, genes indicative of glycolytic metabolism and TGFβ signalling. The transition from naive to primed pluripotent gene expression was further evidenced by the 3138 DEGs between ICM and Sph EPI (Fig. 5a, b, Source data file). GO enrichment and KEGG pathways analyses between these stages showed that PI3K-Akt, Jak-Stat and Interleukin-6-mediated signalling pathways were upregulated in ICM cells (Table 4, Fig. 5b, c).

**Table 4:** Gene ontology and KEGG pathways determined by pairwise comparisons of pluripotent cells of the pig embryo

Lineage	GO terms	KEGG pathways
M vs. EB ICM		
M	Positive regulation of JNK cascade (9.958e−03) Mitochondrial outer membrane permeabilization (1.83e−02) Positive regulation of Wnt signalling pathway, planar cell polarity pathway (1.19e−02) Notch signalling pathway (1.04e−02)	Tight junction (5.67e−11) Hippo signalling pathway (1.11e−06) Wnt signalling pathway (1.11e−05) Notch signalling pathway (2.79e−02) Jak-STAT signalling pathway (3.79e−02)
EB ICM	Endodermal cell differentiation (1.58e−03) Regulation of ERK1 and ERK2 cascade (1.01e−02) Interleukin-6-mediated signalling pathway (4.04e−03) Positive regulation of stem cell population maintenance (6.78e−03)	PI3K-Akt signalling pathway (1.51e−08) Signalling pathways regulating pluripotency of stem cells (3.07e−05) MAPK signalling pathway (3.62e−02)
EB ICM vs. LB EPI		
EB ICM	Positive regulation of interleukin-6 production (2.51e−03) Interleukin-6-mediated signalling pathway (3.16e−03) Hydrogen ion transmembrane transport (4.00e−03) Tricarboxylic acid cycle (8.77e−03) Mitochondrial electron transport, cytochrome *c* to oxygen (1.63e−02)	Signalling pathways regulating pluripotency of stem cells (4.45e−06) PPAR signalling pathway (6.62e−05) PI3K-Akt signalling pathway (1.54e−04) Jak-STAT signalling pathway (2.30e−02)
LB EPI	Regulation of ERK1 and ERK2 cascade (6.76e−04) Response to hypoxia (3.06e−03) Positive regulation of MAP kinase activity (5.37e−03) DNA methylation (1.32e−02) C-5 methylation of cytosine (1.94e−02) Transforming growth factor beta receptor signalling pathway (2.25e−02)	Gap junction (8.74e−04) AMPK signalling pathway (4.62e−03) Axon guidance (2.21e−02) TGF-beta signalling pathway (3.60e−02)
LB EPI vs. Sph EPI		
LB EPI	Regulation of DNA methylation (2.01e−03) Chromatin organisation (2.33e−03) Positive regulation of stem cell proliferation (4.54e−03) Histone H4 deacetylation (2.20e−02)	
Sph EPI	Axon development (2.36e−02) Response to hypoxia (6.50e−03) Negative regulation of apoptotic process (2.94e−03) Cellular response to oxidative stress (1.22e−02)	Focal adhesion (3.88e−04) ECM–receptor interaction (1.59e−02)

Significance values in brackets

*M* morula, *EB* early blastocyst, *LB* late blastocyst, *Sph* spherical embryo, *ICM* inner cell mass, *EPI* epiblast

Interestingly, no significant changes in signalling pathways affecting pluripotency were observed between early (LB) and late (Sph) EPI, indicating that the primed EPI stably maintains its properties over ~6 days (Table 4, Fig. 5d).

### Novel surface specific markers in pig pluripotent cells

**Fig. 6:**
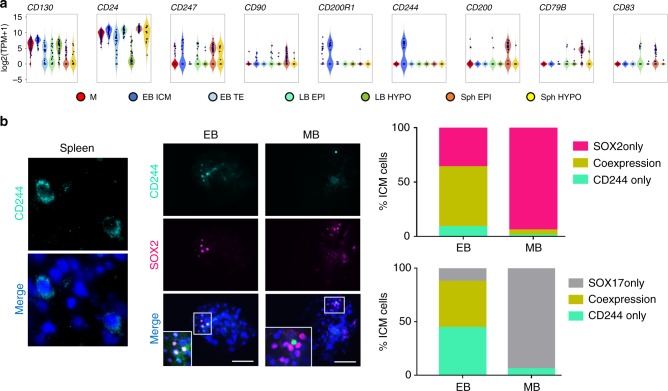
Surface markers of pig pluripotent cells. **a** Expression of surface markers in pluripotent lineages. **b** IF analysis of CD244 in spleen macrophages (positive control) and in early-(EB, *n* = 12) and mid-blastocysts (MB, *n* = 5) (scale bar: 10 µm in spleen; 100 µm in embryos). Bar charts showing the proportion of cells within the ICM expressing CD244 only, SOX2 only, and SOX17 only or co-expressing these markers. M: morula, EB: early blastocyst, LB: late blastocyst, Sph: spherical embryo, EPI: epiblast, HYPO: hypoblast, ICM: inner cell mass, TE: trophectoderm

We sought to identify novel pluripotency markers in the pig, by comparing our scRNA-Seq dataset with the cell surface protein atlas^30^. Surface markers described in human naive and primed pluripotent cells^31^, such as CD130 (IL6ST) and CD24, were not lineage-specific in the pig embryo (Fig. 6a). Instead, we found CD247 primarily marking the ICM and LB EPI, while CD90 (THY1) was detected in LB and Sph EPI (Fig. 6a). Notably, CD200, CD79B and CD83 were specifically expressed in late epiblast cells and could constitute primed pluripotency cell surface markers in the pig. Candidates for new naive markers were CD200R1, expressed only in M and ICM cells, and CD244, expressed exclusively in ICM. We confirmed the expression of CD244 by IF, which unexpectedly showed nuclear localisation within a subpopulation of SOX2+ cells in the ICM of EB. A small number of cells also showed SOX17 co-expression, suggesting that cells segregating towards hypoblast gradually lose CD244 protein. By the MB stage, CD244 was almost undetectable, consistent with scRNA-seq data showing downregulation of this marker in late blastocysts (Fig. 6b).

### Distinct metabolic and epigenetic landscapes of pluripotency

**Fig. 7:**
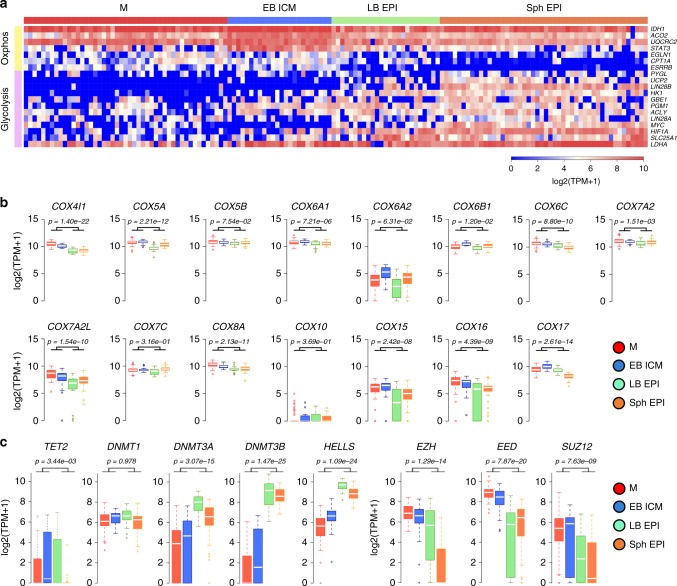
Metabolic and epigenetic changes during progression of pluripotency. **a** Heat map of selected genes involved in OXPHOS and anaerobic glycolysis in pluripotent lineages. **b** Box plot showing expression of electron transport complex genes and **c** genes involved in epigenetic modifications. Boxes show 25–75 percentile values and white line shows median gene expression (*p* < 0.05 by two-sided Wilcoxon test). M: morula (*n* = 47 cells), EB: early blastocyst (*n* = 24 cells), LB: late blastocyst (*n* = 25 cells), Sph: spherical embryo (*n* = 48 cells), EPI: epiblast, ICM: inner cell mass

A shift towards glycolytic metabolism and reduced mitochondrial activity is associated with the development from naive to primed pluripotency in mouse and human PSC^32,33^; this metabolic switch has also been described in the mouse epiblast^33^. DEG and GO term analysis between ICM and Sph EPI cells suggested a metabolic switch during this period in the pig embryo (Table 4). Notably, *ESRRB* and *STAT3*, which stimulate oxidative phosphorylation (OXPHOS) during maintenance of naive pluripotency^34^, were upregulated in M and ICM, but were later downregulated in LB and Sph EPI. Enzymes involved in the tricarboxylic acid (TCA) cycle and OXPHOS, such as *IDH1*, *ACO2* and *UQCRC2* followed the same trend, as well as *EGLN1*, which prevents HIF-1α stabilisation and is downregulated in primed pluripotent cells^35^ (Fig. 7a; Supplementary Figure 3). *LIN28A* and *LIN28B* maintain low mitochondrial function in primed pluripotent cells^32,36^, and *MYC* binds to the *LIN28B* locus and potentiates glycolysis^37^. These genes were upregulated in pig EPI. A similar expression pattern was noted for *HIF-1α*, a hypoxia-inducible factor upregulated during the transition from naive to primed state^33^, concomitantly with the upregulation of downstream enzymes *HK1*, *GBE1*, *PGM1* and *PYGL*, required to convert glucose to glycogen. Finally, the glycolytic enzymes *LDHA*^33,38^ and metabolite transporter *UCP2*, which limit pyruvate oxidation and facilitate glycolysis, were also upregulated in EPI cells (Fig. 7a; Supplementary Figure 3). We also detected a reduction in expression of electron transfer complex IV (cytochrome *c* oxidase) genes (11/20 genes) during the maturation of the epiblast (Fig. 7b), suggesting a reduction in mitochondrial metabolism^33^.

We observed downregulation of the fatty acid transporter to the mitochondria *CPT1A* and a concomitant increase of critical fatty acid synthesis genes *SLC25A1* and *ACLY* in EPI cells compared to ICM; this is in agreement with previous reports indicating accumulation of long-carbon-chain lipids during the conversion from naive to primed pluripotency in mouse and human^35^ (Fig. 7a).

Epigenetic modifications are highly responsive to metabolites derived from pathways such as the TCA cycle or glycolysis, in particular, DNA methyltransferases (DNMT), histone acetyltransferases and histone methyltransferases^39^. GO terms related to de novo DNA methylation were upregulated in EPI cells (Fig. 5c, Table 4). Accordingly, the expression of *DNMT3A*, *DNMT3B* and *HELLS*, required for de novo DNA methylation^40^, significantly increased in LB and Sph EPI. Concomitantly, *TET2* was downregulated in the late EPI (Fig. 7c).

The core components of PRC2 complex *EZH2*, *EED* and *SUZ12* repress developmental regulators through establishing trimethylation of lysine 27 in histone 3 (H3K27me3) modification^36^, preventing differentiation of PSCs^41^. These genes were expressed at all stages harbouring pluripotent cells in the pig embryo, while expression of *EZH2* and *EED* was downregulated in primed pluripotent stages (Fig. 7c), similar to previous observations in pig epiblasts^42^ and human PSCs^35^. Hence, two populations of pluripotent cells with distinct metabolic and epigenetic profiles exist in the early pig embryo.

### Dosage compensation of X-chromosome in pig embryos

**Fig. 8:**
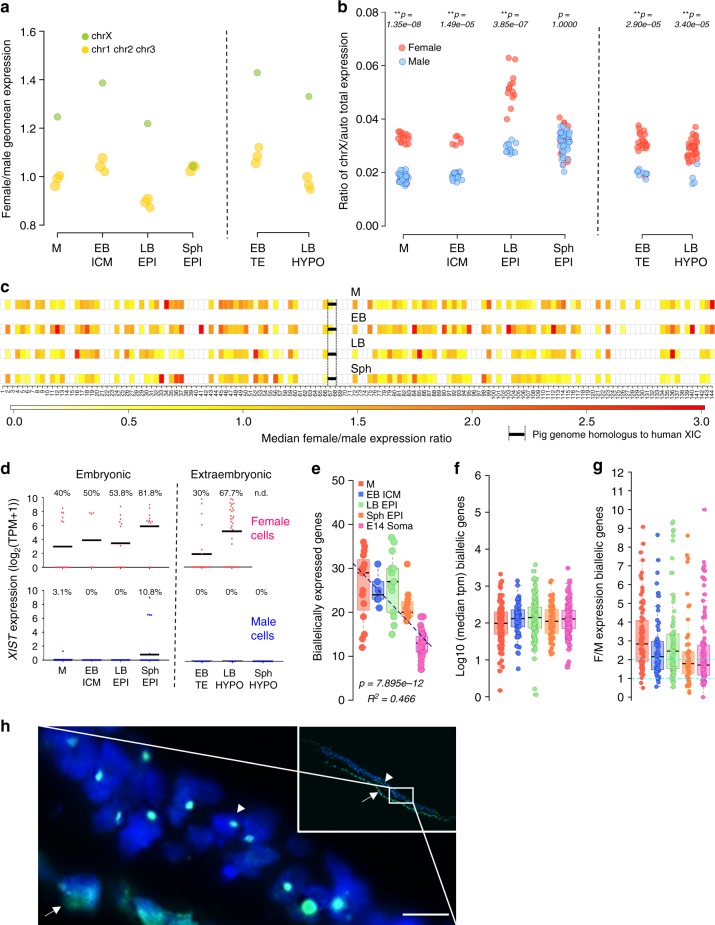
Dosage compensation for the female X-chromosome. **a** Ratio of gene expression between female and male embryos for the X-chromosome vs. autosomes 1, 2 and 3. **b** Proportion of total expression levels of the X-chromosome relative to autosomes at the single-cell level. **p ≤ 0.01, two-sided Wilcoxon test. **c** Female-to-male expression average along the X-chromosome. XIC: X-inactivation centre. **d** *XIST* expression level in male and female cells (black line indicates mean expression). Percentage of cells with TPM > 1 is shown. **e** Number of biallelically expressed genes in each cell at different stages of development. Linear regression used to determine trend line. **f** Median expression of bi-allelic genes. **g** Female-to-male ratio of expression of genes biallelically expressed in females. Boxes show 25–75 percentile data points and black line shows median values (**e**–**g**). Light blue lines (**g**) depict values 1 and 2 across the dataset. **h** IF staining of H3K27me3  (green) merged with DAPI (blue)  in sectioned spherical female embryo. Arrow indicates hypoblast and arrowhead marks the epiblast. Inset shows a low magnification image of the embryonic disc. Scale bar: 10 µm. M: morula, EB: early blastocyst, LB: late blastocyst, Sph: spherical embryo, EPI: epiblast, ICM: inner cell mass, HYPO: hypoblast

To establish the gender of each cell/embryo, the cumulative level of Y chromosome gene expression was established (Supplementary Figure 4a-c). The female-to-male ratio of X-chromosome (XC) gene expression was higher in females from morula to LB in all embryonic lineages, suggesting lack of dosage compensation. However, in Sph EPI, XC gene expression was comparable to that of autosomes in all embryos, indicating the occurrence of dosage compensation (Fig. 8a). Analysis of XC gene expression relative to autosomes at the single-cell level showed uniformity between male and female cells and confirmed dosage compensation in Sph EPI (Fig. 8b). A chromosome-wide analysis of female-to-male ratio showed a progressive reduction in gene expression along the whole chromosome with some areas maintaining high ratios of expression at the spherical stage (Fig. 8c). In agreement with the dynamics of dosage compensation, *XIST* expression was detected in most (81.8%) female cells in the EPI of Sph embryos, with only sporadic expression of *XIST* in some male cells (Fig. 8d).

To investigate the mechanism of dosage compensation, we analysed XC expression at an allelic resolution, quantifying the expression of single nucleotide variants (SNV) within each cell for a reference or an alternative allele. As expected, SNVs were not found in male cells, consistent with the presence of a single XC (Supplementary Figure 4d). Notably, there was a sharp decline in the number of biallelically expressed genes in spherical EPI. The lowest level was detected in female mesoderm cells from E14 embryos, where we detected an inactive XC (Fig. 8e; Supplementary Figure 4d), which served as a somatic cell control. This result indicates that dosage compensation at the spherical stage is attained by inactivation of one XC. To gain a better understanding of the X-inactivation process, we analysed the median expression of biallelically expressed genes. No median reduction in bi-allelic gene expression was detected en route to dosage compensation (Sph EPI) (Fig. 8f). The female/male ratio of biallelically expressed genes was close to 2 in the stages showing dosage compensation (Fig. 8g). This result suggests that “dampening” of X-linked gene expression does not precede dosage compensation. To confirm the inactivation of one X-chromosome in the epiblast of female spherical embryos, we analysed Histone H3 lysine 27 trimethylation (H3K27me3), which accumulates in the inactive X^43^. A clear single focal enrichment of H3K27me3 was detected in the nuclei of epiblast cells in female spherical embryos (Fig. 8h), similar to what is observed in mesodermal cells (Supplementary Figure 4d). In contrast, no H3K27me3 foci were found in female LB cells, consistent with the lack of XCI (Supplementary Figure 4d).

## Discussion

We revealed the molecular features of early lineage segregation, pluripotency and X-inactivation during development of early pig embryos. Our study provides the basis for comparisons with human and mouse development, and for insights in conservation and divergence of early mammalian development.

Segregation of the first three lineages occurs progressively during pre-implantation development, starting with the TE and the ICM in early blastocysts. High levels of *GATA2* and *GATA3* expression detected in early pig TE cells conform to the observations in early human and *Cynomolgus* monkey TE^2,17^. By contrast, *Cdx2* expression is among the earliest markers in the mouse TE^44,45^. ICM cells of early pig blastocysts co-express EPI (*NANOG, SOX2*) and HYPO (*GATA6, PDGFRα, SOX17*) markers, but during the mid-/late blastocyst stage EPI and HYPO lineages become definitively segregated, indicating that ICM cells are bi-potent, able to give rise to mature EPI and HYPO, as shown in mouse^46^, human^23^ and monkey^2^.

Pathway analysis revealed Jak-Stat signalling enrichment in TE and ICM of early embryos. The Jak-Stat pathway is an effector of multiple ligand/receptor interactions including members of the IL6 family, such as LIF, GCSF and IL6. A previous study showed that Jak-Stat signalling is essential for TE development in the pig^25^. Although there is expression of *LIFR* in some ICM and TE of early blastocysts, there is no expression of *LIF*; either in any of the cells of the blastocyst, or in the maternal endometrial cells at this stage^47^, suggesting that LIF signalling does not have a significant role in early pig embryos. Instead, expression of *IL6* in morulae and TE cells of EB, and *IL6R* and the co-receptor *IL6ST* (also known as GP130) in ICM and TE cells, suggests that IL6 likely activates the Jak-Stat pathway by binding to its cognate receptor. IL-6 supplementation had a modest effect in promoting TE proliferation, and did not affect ICM development, in contrast to a previous report on parthenogenetic pig embryos^48^. However, *IL6*^−/−^ gene-edited embryos had a reduced number of TE cells, but unaffected ICM. Given that blocking Jak-Stat signalling results in a reduction in TE cells, these experiments show that Jak-Stat signalling via IL6 has a critical role during pig TE specification. Since Jak-Stat signalling can be stimulated by other pathways^49^, such as PI3K-AKT and MAPK, which we showed can affect the number of ICM cells when blocked, the effects of Jak inhibition on the ICM may be indirect and not specific to IL6 signalling.

Signalling via MEK is important for hypoblast formation in mouse^26^ and rabbit^27^; though this does not seem to be the case in human^23^, marmoset^13^, pig^25^ and cattle^24^. Only when using high concentrations of MEK inhibition we detected a drastic decrease in SOX17 expression, as reported in cattle^28^, suggesting that alternative pathways may be supporting HYPO segregation in large mammals.

Our study reveals that TGFβ signalling is critical during the expansion of the epiblast between MB-LB transition, but not in EPI/HYPO segregation, consistent with previous reports^24,25^. TGFβ signalling is needed for hESC self-renewal^50^, and inhibition of this pathway affects NANOG expression in human and marmoset blastocysts^11,13^. Similarly, NANOG expression in pig embryos is also affected by inhibition with SB431542. Furthermore, we show high expression of TGFβ components in EPI cells compared to ICM, suggesting that this pathway becomes active in advanced embryos, pointing to a critical role of TGFβ during the epiblast expansion.

Analysis of embryonic cells revealed an expression profile with genes characteristic of naive pluripotency in morula and ICM cells (made of ~10–15 cells) of EB (E~5–6), and of primed pluripotency in the EPI of LB (~E7/8) and Sph (~E10/11) embryos, which coincides with an expansion of the epiblast from ~25 cells in LB to more than ~180 in Sph. Given that cells with naive pluripotent gene expression are only transiently present (~1 day) and instead cells with primed pluripotent expression grow for a protracted period (~3–4 days), the latter could be captured as self-renewing cells in vitro. Indeed, pig pluripotent cell lines with primed characteristics have been reported^21,51–53^, but not of those with characteristics of naive pluripotency; the latter may require different culture conditions capable of stimulating their proliferation. Differences in naive pluripotency properties between mouse and other mammals may underlie the difficulties in establishing equivalent cells from the latter in vitro. Pig naive pluripotency markers include *KLF4/5/17*, *TBX3* and *TCFPL21*, and are consistent with those reported in human^11^ and monkeys^2,13^, which differ from mouse naive pluripotency, which is characterised by the expression of *Klf2*, *Prdm14* and *Bmp4*. These genes participate in regulating gene expression, epigenetic reprogramming, and cellular signalling, respectively, which highlight potential functional differences in naiveté between mice and larger mammals.

The transition from naive to primed pluripotent gene expression in the pig embryo is accompanied by a change in expression of genes indicative of a metabolic shift from OXPHOS towards glycolysis. This is supported by an increase in l-lactate production in late pig blastocysts compared to morulae^54^. The metabolic switch likely provides critical metabolites to promote epiblast expansion, as well as epigenetic remodelling through epigenetic modifications of DNA and histones, a crucial step in preparation for the next major developmental event that is the onset of gastrulation.

Diverse mechanisms exist in mammals for dosage compensation with respect to the XC in females^55^. In mice, imprinted XCI results in inactivation of the paternal XC in early cleaving embryos, followed by reactivation in the ICM of blastocyst (excluding the extraembryonic tissues), and then random XCI in the epiblast^56^. In contrast, there is no imprinted XCI in human and rabbit embryos. Indeed, the expression of *XIST* from both X chromosomes in blastocysts suggests alternative mechanisms of dosage compensation^56^. The “dampening” of X-linked genes from both parental chromosomes as a possible mechanism^12^ warrants further studies^57^. Another report indicated incomplete dosage compensation of a subset of X-linked genes in pig blastocysts^15,58^. Notably, our observations however show XCI in the mature EPI, as demonstrated by the reduction in the number of biallelically expressed X-linked genes, coupled with the appearance of the H3K27me3 mark on the inactive XC.

**Fig. 9:**
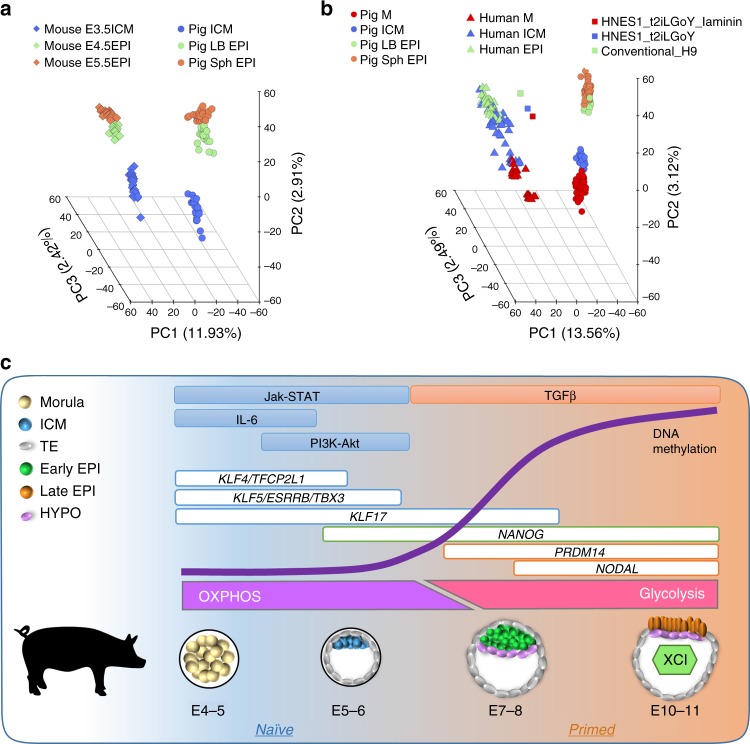
Comparison of pig, mouse and human matched pluripotent states. **a** PCA of pig and mouse orthologous genes expressed in pluripotent cells. **b** PCA of pig and human orthologous genes expressed in embryonic cells and hESCs. **c** Summary of key events in the pluripotent compartment of the pig embryo. E: embryonic day, M: morula, EB: early blastocyst, LB: late blastocyst, Sph: spherical embryo, EPI: epiblast, HYPO: hypoblast, ICM: inner cell mass, TE: trophectoderm

Our study at the resolution of single cells allows comparisons between species to identify developmental equivalence. Comparison of mouse and pig pluripotent matched stages showed broad developmental alignment, although the developmental time in mice is three times shorter compared to pigs (2 days vs. 6 days). Yet the overall principles of the emergence and establishment of pluripotency are conserved between these species (Fig. 9a). Developmental progression showed broad equivalence between morula to epiblast transitions in humans and pigs. Importantly, human embryonic stem cells (hESC) with naive and primed characteristics grouped closely to human late ICM and EPI cells, respectively, and these also aligned with pig EPI cells (Fig. 9b). Our observations may be relevant for understanding events during early human development, as well as for attempts to study specification of hESCs in chimeras with pig embryos as hosts, following their introduction into blastocysts. Hitherto, the reported efficiency of these experiments is very low^59^, perhaps because the hESCs were not in-sync with the host pig blastocyts. Developmental synchrony between donor and host is important for efficient chimerism^60^. We propose that the introduction of primed state hESCs into late pig blastocysts may be a more favourable environment for homing of hESC, and their subsequent development in chimeras.

In conclusion, this comprehensive analysis depicts molecular landmarks of pig embryogenesis that provides new insights into embryos with protracted epiblast development (Fig. 9c). Furthermore, the shared features of lineage segregation and pluripotency between humans and pigs revealed here will help accelerate research into novel approaches in regenerative medicine, such as the development of interspecies chimeras.

## Methods

### Porcine embryo collection

All of the procedures involving animals have been approved by the School of Biosciences Ethics Review Committee, The University of Nottingham. Embryos at each stage were retrieved from multiple crossbred Large White and Landrace sows (2–3 years old) between days 4 and 11 after artificial insemination. Embryos were flushed from the uterine horns with 30–40 ml warm PBS (supplemented with 1% FCS), washed and transported to the laboratory in N2B27 supplemented with 25 mM HEPES in a portable incubator at 38.5 °C.

### Oocyte collection and IVM

Oocytes were aspirated from antral follicles (3–6 mm diameter) of ovaries from pre-pubertal gilt ovaries collected at a local slaughterhouse. Cumulus-oocyte complexes (COCs) with several layers of unexpanded cumulus cells and evenly dark cytoplasm were selected for maturation. COCs were washed in TCM-199 containing 2 mg ml^−1^ BSA, and placed in individual wells containing 500 μl of maturation medium composed of TCM-199 containing 3.05 mM glucose, 0.91 mM sodium pyruvate, 0.57 mM cysteine, 0.1% w/v polyvinyl alcohol, 10 ng ml^−1^ EGF, 20 ng ml^−1^ LIF, 40 ng ml^−1^ FGF2, 10 ng ml^−1^ FSH and 0.5 µg ml^−1^ LH for 42–44 h at 38.5 °C in 5% CO_2_ air^61^.

At the end of the maturation period, the cumulus cells adhering to the oocyte were removed by pipetting for 2 min in 1 mg ml^−1^ hyaluronidase. Matured oocytes were identified by the presence of a polar body.

### sgRNA design and in vitro transcription

The online software MIT CRISPR Design Tool (http://crispr.mit.edu) was used to design sgRNAs targeting *IL6* gene. The CRISPR/Cas9 target sequences (20 bp target and 3 bp PAM sequence (underlined)) used in this study are shown as follow: gRNA1: ATCTTCTTCCAGGCGTCCCGGGG; gRNA2: TCATTGCAGAGATTTTGCCGAGG. The sgRNAs were produced using Geneart Precision gRNA Synthesis Kit (Thermo Fisher Scientific) and specific primers (Supplementary Information). sgRNAs were tested individually and in combination in parthenogenetic embryos.

### Microinjection of sgRNAs and Cas9 protein

Matured oocytes were washed with TCM-199 containing 25 mM Hepes (Gibco), transferred into a 30 μl drop of TCM-199-Hepes medium and placed on an inverted microscope fitted with micromanipulators to microinject Cas9 ribonucleoprotein complex mixture. Briefly, 3 μl of 250 ng μl^−1^ Cas9 protein (ToolGen Inc, South Korea) was mixed with 3 μl of 250 ng μl^−1^ sgRNA mixture (gRNA1 and gRNA2) and incubated for 10 min at 37 °C. The resulting Cas9 ribonucleoprotein complex mixture was diluted to a final concentration of 25 ng μl^−1^ for microinjection. Cas9 ribonucleoprotein complex was loaded to a spike-end micropipette of 5–7 μm internal diameter (ID) connected to a manual hydraulic air microinjector. Zygotes were secured by a holding pipette and the pipette was advanced into the zygote, and cytoplasm was aspirated until the plasma membrane was broken to then deliver the Cas9 ribonucleoprotein complex into the cytoplasm. Groups of 20 zygotes were manipulated simultaneously. After microinjection, the oocytes were parthenogenetically activated.

### Parthenogenetic activation and embryo culture

Microinjected oocytes were electrically activated with two pulses of 120 V mm^−1^ for 40 μs, delivered by an Eppendorf Multiporator using a 0.5 mm chamber containing 0.3 M mannitol, 0.05 mM CaCl_2_, 0.1 mM MgSO_4_ and 0.1% bovine serum albumin (BSA). After washing with PZM5, the oocytes were chemically activated with 2 mM DMAP and 5 μg ml^−1^ cytochalasin B in PZM5^62^ for 5 h to generate diploid parthenogenetic embryos. After activation, porcine zygotes were cultured in 500 μl of PZM5 containing 0.3% BSA for 5 days. After 5 days of culture, embryos were cultured in N2B27 supplemented with 0.3% BSA^25^ at 38.5 °C in a humidified atmosphere of 5% CO_2_, 5% O_2_ and 90% N_2_. Day 7 blastocysts were fixed in 2% paraformaldehyde (PFA) for 10 min at RT after removing zona pellucidae with Tyrode’s acid. After immunostaining embryos were recovered for genotyping.

### Embryo genotyping after gene editing

Each embryo was placed in 2.5 µl Accutase, and 5 µl Alkaline Lysis buffer (50 mM NaOH, 0.4 mM EDTA) was added. After 20 min incubation at 99 °C, the lysis reaction was neutralised with 5 µl 40 mM Tris–HCl (pH 5). DNA was amplified in two rounds of 40 cycles of PCRs using KAPA HiFi HotStart ReadyMix (Roche) for the first round and Sigma ReadyMix with 1:10 of the first PCR product for the second round and specific primers (Supplementary Information). Agarose gel electrophoresis was followed by gel extraction of the bands and Sanger Sequencing. The sequences were aligned to the Wildtype sequence using the MUSCLE alignment tool (EMBL-EBI). For mixed pherograms, the TIDE webtool was used^63^.

### Isolation of single cells for single-cell cDNA preparation

Zona pellucidae were removed using acidic Tyrode’s solution (Sigma) in morulae and early blastocysts, and then embryos were dissociated. Late blastocysts were subjected to immunosurgery to remove the trophectoderm based on previously described procedures^64^. Briefly, embryos were incubated for 30 min in a 1:5 dilution of anti-pig serum (Sigma) in N2B27 medium, washed and incubated for 30 min in 1:5 dilution of complement (Sigma). Embryos were transferred to N2B27 for a few minutes for efficient cell lysis, and then embryonic disks were isolated from the trophectoderm by repeated aspiration with a pulled glass capillary. In spherical embryos, epiblast and hypoblast were manually isolated. Trophectoderm cells were not collected from late blastocysts and spherical embryos.

Single-cell dissociation was performed by incubation in TrypLE Express (GIBCO) for 5 min at 37 °C and repeated pipetting using very thin pulled capillaries. Individual cells were subsequently transferred to DMEM +20% FCS to block TrypLE Express and washed in a small drop of PBS-PVP. Single cells were manually collected into PCR tubes to prepare single-cell cDNA libraries following the Smart-seq2 protocol^65^.

Briefly, single cells were lysed by incubation at 72 °C for 3 min in PCR tubes containing 4 μl of cell lysis buffer, oligo-dT primer and dNTP mix. Reverse transcription and PCR pre-amplification were carried out with SuperScript II (Invitrogen) and KAPA HiFi HotStart ReadyMix (KAPA Biosystems) respectively according to Picelli et al. protocol. PCR products were purified using Ampure XP beads (Beckman Coulter), and library size distribution was checked on Agilent dsDNA High Sensitivity DNA chips on an Agilent 2100 Bioanalyzer (Agilent Technologies). Concentration was quantified using Qubit Quant-iT dsDNA High-Sensitivity Assay Kit (Invitrogen). Samples with more than 0.2 ng µl^−1^, free of short fragments (<500 bp) and with a peak at around 1.5–2 kb were selected for library preparation with Nextera XT DNA Library Preparation Kit (Illumina). Tagmentation reaction and further PCR amplification for 12 cycles were carried out, and PCR products were again purified using Ampure XP beads. Quality of the final cDNA library was analysed on an Agilent high-sensitivity DNA chip. Final cDNA libraries had an average size of 700–800 bp and were quantified using NEBNext Library Quant Kit for Illumina (New England BioLabs) following the manufacturer instructions. Finally, libraries were pooled in groups of 50 with a 2 nM final concentration, and DNA sequencing was performed on a HiSeq 2500 Sequencing System (Illumina).

### Single-cell RNA-Seq data

Raw PE reads were trimmed against adaptor sequences by *scythe* (v0.981), and quality-trimmed by *sickle* (v1.33) using default settings. Trimmed reads were directionally aligned to the pig genome (*Sus scrofa* v10.2) by *hisat2* (v2.1.0) with *-know-splicestie-infile* setting to increase mapping accuracy of splicing reads. Uniquely and correctly mapped reads were extracted for the downstream analysis. *htseq-count* was used to count the number of reads aligned to each gene (*Sus scrofa* v10.2 ensembl annotation build 87). Gene expression level was calculated and normalised by Transcripts Per Kilobase Million (TPM).

Low quality cells were filtered out from the dataset to reduce the downstream analysis noise. First, the total number of reads mapped to gene transcripts was calculated for each cell, and those with less than 1 million were removed. Second, the proportion of reads aligned to mitochondrial genes was estimated, as a high proportion suggests poor quality cells^66^. The proportion cut-off was set at 0.5. Only cells of proportions below 0.5 were kept for the next analysis. Third, 4 outlier cells were identified by t-SNE dimensionality reduction. A total of 13,815 out of 22,824 annotated genes were identified in at least 3 cells with TPM > 1.

### Lineage segregation of cells

The R package “*scater*” was applied to normalise read counts of genes for each good quality cell with acceptable sequencing coverage. A non-linear approach, t-stochastic neighbour embedding (t-SNE), was used to identify the relations between cells using normalised read counts. Unsupervised hierarchical clustering using all expressed genes as input was conducted on all filtered cells by normalised read counts in log_2_ scale. The distance method was *euclidean*, and the cluster method was *ward.D2*.

### Lineage differential expression analysis

Pairwise comparisons of single-cell differential expressions were performed by SCDE using normalised read counts among four embryo stages. Two-tailed adjusted *p*-value were calculated using cZ scores from Benjamini–Hochberg multiple testing corrections, and followed a normal distribution. Significantly expressed genes were selected with a *p*-value <0.05 as the threshold. A heat map of differentially expressed genes (DEGs) was created with a log_2_ scale of normalised expression. *Euclidean* distance and default *hclust* were applied to determine the relationships between cells and between genes. Gene Ontology (GO) gene set enrichment analysis with DEGs utilised *goseq* for each pairwise comparison, also with upregulated DEGs and downregulated DEGs separately. GO term annotation was retrieved from the Ensembl database (*Sus scrofa* v10.2 ensembl annotation version 87). Enrichment analysis of biological pathway was performed with DEGs by *gage*. Ensembl gene IDs of DEGs were mapped to NCBI gene IDs for KEGG pathway prior to enrichment analysis.

### Lineage subpopulation analysis

Cell lineages were investigated for subpopulation analysis. An outlier EB ICM cell was excluded by PCA based on log_2_ TPM values of all expressed genes. LB and Sph cells were grouped together for PCA. Two contrasting methods of PCA were applied; one using all expressed genes, and the other based on the highly variable genes (HVGs) only. We used *decomposeVar* to detect HVGs with a loess regression fit model. FDR ≤ 0.05 was applied as a significance cut-off.

### Inference of embryonic sex

Expressions of all the Y chromosomal genes were summed up to determine the sex of each cell. A cell with the total chromosome Y TPMs ≥ 100 $(\mathop {\sum }\limits_{{\mathrm{gene}}}^{{\mathrm{chrY}}} {\mathrm{TPM}})$ was regarded as a male cell, while $\mathop {\sum }\limits_{{\mathrm{gene}}}^{{\mathrm{chrY}}} {\mathrm{TPM}}$<100 regarded as a female cell.

### Chromosome X dosage compensation analysis

Genes of chromosome X and three autosomes (chr1, chr2, chr3) were extracted, and the geometric mean TPM of chromosomal expressed genes was calculated for each cell separately. Then the overall geometric mean TPM was obtained for each developmental stage by embryo sex, as well as the total TPM. Each TPM value was incremental by one (TPM + 1) for the calculation of geometric mean TPM. Only shared expressed genes between female and male cells were taken into account in the calculation of female/male expression ratio for each chromosome. For each cell, the ratio of chrX/auto was inferred by total TPMs, and grouped by embryo sex. Median Female/Male expression ratio was estimated for each stage across the whole chromosome X with 1 Mb window.

### Analyses of allelic expression

Trimmed reads were aligned to chromosome X of the pig genome (*Sus scrofa* v10.2) by *hisat2*. Duplicated reads were marked by *picard* (v2.12.1). GATK (v3.8) was used to retrieve allelic read counts for SNVs annotated in dbSNP (build 147). Only validated SNVs (dbSNP flag VLD) were extracted for downstream analysis. SnpEff (v4.3) was applied to annotate called SNVs with *Sus scrofa* v10.2 ensembl annotation (build 87). Low coverage SNVs (<3 reads) were excluded from the analysis, and we only kept SNVs that occurred at least in two different cells for each stage. The expressions of mono-/bi-allelic genes were estimated based on SNVs in each female cell of each stage.

### Gene clustering by expression profile

Self-organising map (SOM) were used to discover potential structural patterns in highly dimensional and complex datasets by creating 2-dimensional representations. The SOM algorithm was applied to our gene expression profile. The geometric mean TPM of each gene was calculated within each stage. In order to fit the SOM model, the lower TPMs (<1) were replaced by 1, and the extreme higher TPMs were replaced by 10,000. Similarly, genes of highly similar expression profiles within stages were excluded $(\frac{{{\mathrm{max}}({\mathrm{TPM}})}}{{{\mathrm{min}}({\mathrm{TPM}})}} \le 1\;{\mathrm{or}}\max \left( {{\mathrm{TPM}}} \right) - \min \left( {{\mathrm{TPM}}} \right) \le 0)$. The filtered TPMs were then normalised by SOM (*μ* = 0, *σ* = 1). In total, 25 clusters were created.

### Comparison of pig, mouse and human datasets

In total, 144 pig cells (our study), 83 mouse^8^ cells and 152 human cells retrieved from Petropoulos et al.^12^ and re-classified according to Stirparo et al.^7^ were included in the comparative analysis. PSC lines cultured under conventional or naive conditions were used for analysis as described by Stirparo et al.^7^ Pig orthologous genes (15,171) were retrieved against human genes from Ensembl database (compara build 87). Expression values were normalised by TPM for PCA analysis. Linear regressions were calculated separately for PC2 and PC3, which contributed to pig and human developmental genes, respectively.

### Embryo treatments with inhibitors, growth factors and IF

Embryos were incubated in PZM5 culture medium up to morula stage and in N2B27 medium supplemented with 0.3% fatty acid free BSA from compact morula onwards, in a humidified atmosphere at 39 °C and 5% O_2_. The embryos were treated with the following inhibitors and cytokines: 10 µM PD0325901 (Tocris), 20 µM SB431542 (Tocris), 10 µM LY294002 (Selleckem), 2.5 µM IWP2 (Sigma), 10 µM AZD1480 (Sigma), 10 ng ml^−1^ porcine IL-6 (R&D), 10 ng ml^−1^ TGFβ1 (Peprotech). All inhibitor treatments were performed for 48 h during the indicated time points; from pre-morula (PM) to early blastocyst (EB), from morula (M) to mid blastocyst (MB) and from mid blastocyst to late blastocyst (LB). For the growth factors embryos were cultured from the morula to the late blastocyst stage (72 h). Inhibitors were dissolved in DMSO and control embryos were treated with DMSO accordingly.

After the treatments, embryos before hatching stage were treated with Tyrode’s acid to remove zona pellucidae. Then, embryos were fixed in 4% paraformaldehyde (PFA) for 15 min at room temperature (RT), washed in PBS-1% BSA, permeabilized in 0.2% Triton X-100 for 15 min at RT and blocked in blocking solution (PBS with 0.1% BSA, 0.2% Tween and 10% Donkey serum) for 1 h at RT. Embryos were incubated overnight at 4 °C with the primary antibodies: NANOG (Peprotech, 500-P236, 1:200 dilution in blocking solution), SOX17 (R&D, AF1924, 1:200), SOX2 (Santa Cruz, sc-17320, 1:300), CD244 (Proteintech, 16677-1, 1:100), H3K27me3 (Active Motif, 39157, 1:500). After 4 washes in PBS-1% BSA, embryos were incubated in the appropriate secondary antibodies: Donkey anti-rabbit IgG Alexa 488 (Invitrogen, R37118, 1:500), Donkey anti-goat IgG Cy3 (Jackson, 705-165-003, 1:500) for 45 min at RT, followed by four washes in PBS-1% BSA. Finally, embryos were mounted in Fluoroshield with DAPI (Sigma).

### Statistical analysis

To evaluate the statistical differences in cell count numbers from individual embryos, probability (*p*) values were calculated using two-sided Mann–Whitney test between each treatment and the control. Percentages of contribution of NANOG+ only, SOX17+ only and co-expressing cells were evaluated by two-way ANOVA (Dunnett’s multiple comparisons test). Differences were considered significant when *p* < 0.05.

### Reporting summary

Further information on experimental design is available in the Nature Research Reporting Summary linked to this article.

## Supplementary information

Supplementary Information

Peer Review File

Reporting Summary

Source Data

## Data Availability

All data generated or analysed during this study are included in this published article and its supplementary information files. The source data underlying Figs. 1a, 2a–d, 3c-f, 6d, h and 7c and Supplementary Figures 1a and 5d are provided as a Source Data file. The scRNA-Seq datasets generated during this study are available under GEO accession number: GSE112380 .
