# Supplementary material for: Pluripotency and X chromosome dynamics revealed in pig pre-gastrulating embryos by single cell analysis
Source: Nat Commun. 2019 Jan 30;10:500. doi: 10.1038/s41467-019-08387-8 (PMC6353908; doi:10.1038/s41467-019-08387-8)
Supplement: Supplementary file 1 — Supplementary Information [file 41467_2019_8387_MOESM1_ESM.pdf]

Supplementary Information

Pluripotency and X chromosome dynamics revealed in pig pre-gastrulating embryos by single cell analysis

Ramos-Ibeas et al.

Supplementary Table 1. Primers list

**Primer name**	**Sequence**
*IL6*_F	CTTCAGTCCAGTCGCCTTCT
*IL6*_R	TCCAGCCCCGCAGTATATTT
*IL6*_gRNA1_F	TAATACGACTCACTATAGATCTTCTTCCAGGCGTCCCG
*IL6*_gRNA1_R	TTCTAGCTCTAAAACCGGGACGCCTGGAAGAAGAT
*IL6*_gRNA2_F	TAATACGACTCACTATAGTCATTGCAGAGATTTTGCCG
*IL6*_gRNA2_R	TTCTAGCTCTAAAACCGGCAAAATCTCTGCAATGA

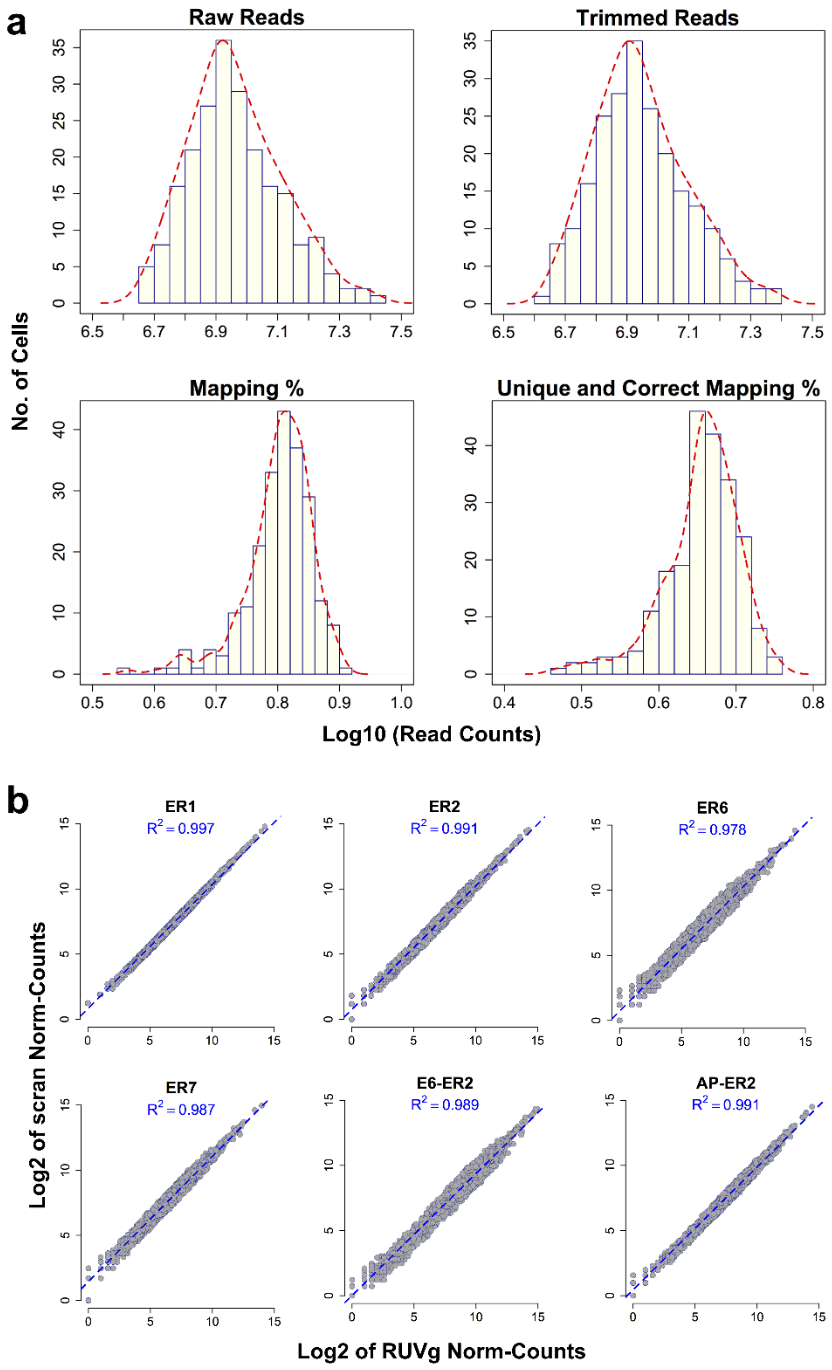

Supplementary Figure 1. Samples and quality control. a, Histogram summaries of sequenced cells with read counts. Both average raw read count and trimmed read counts were ~8 million. Mapping rates of most cells ranged from 70% to 90%; while unique and correct mapping rates were from 65% to 75%. **b,** 6 pig cells included ERCC control. Comparison between RUVg normalisation by ERCC and single cell size-factor normalisation suggested high similarity between two methods. RUVg uses negative control genes to estimate the factors of unwanted variation. In our analysis, we used k = 2 and dropped = 1 in RUVg. E: embryonic day.

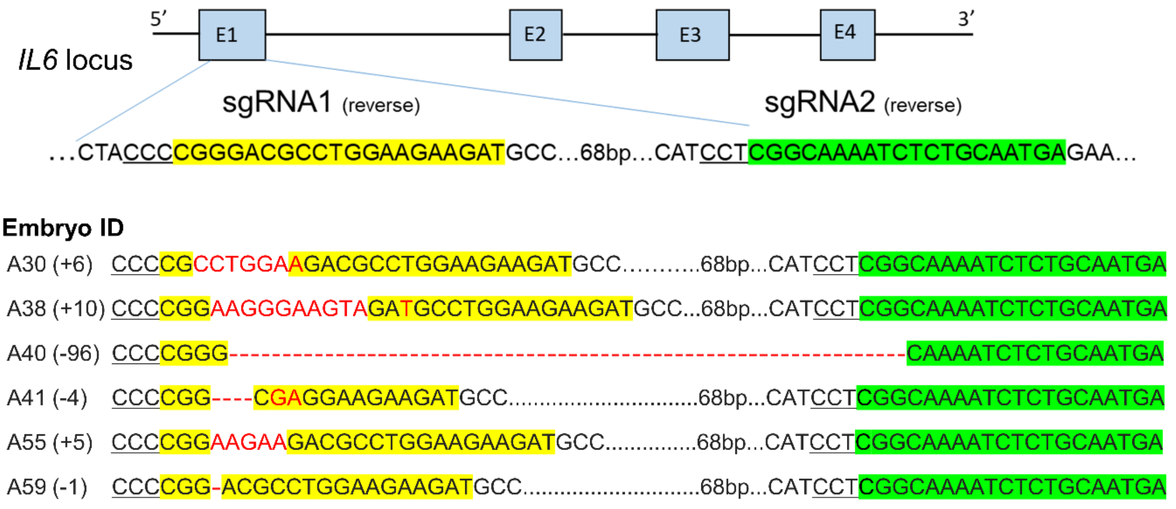

Supplementary Figure 2. Schematic diagram of the location and sequences of the two sgRNAs designed to target the exon 1 of *IL6* gene. Guides are highlighted in yellow and green. Sanger sequencing results from representative homozygous *IL6*$^{-/-}$ blastocysts show a variety of INDELS. Underlined letters indicate the PAM sequence. Red dashes represent deletions; red letters indicate insertions. Embryo ID and base pair insertions (+) or deletions (-) are indicated in brackets.

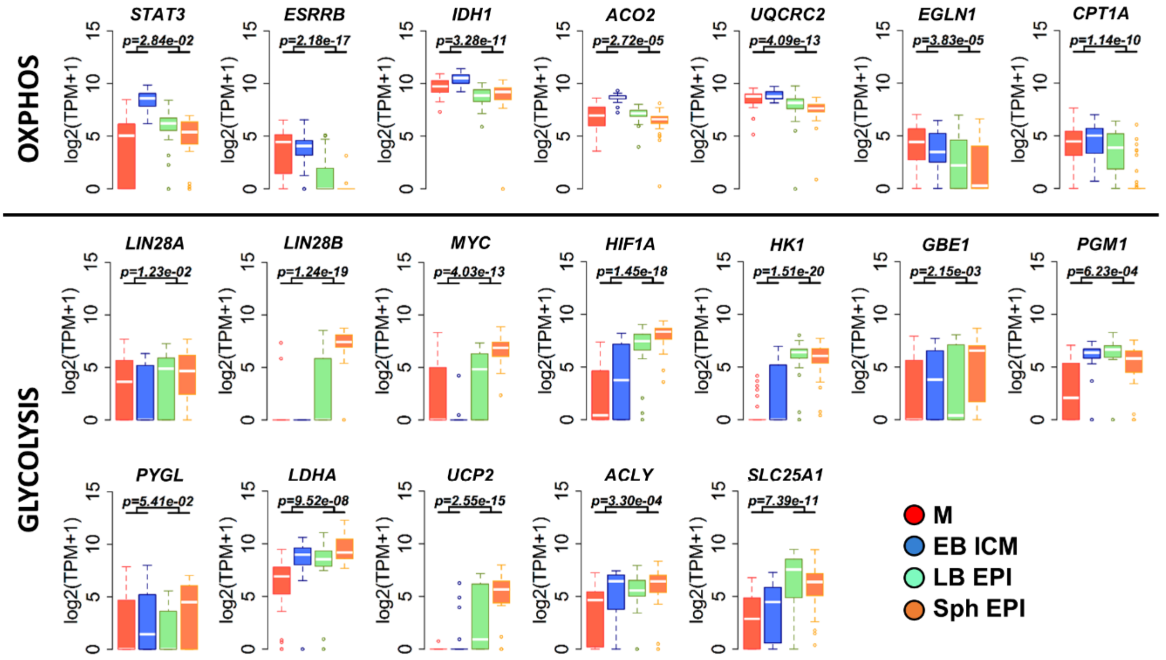

Supplementary Figure 3. Expression of metabolic genes in early pig embryos. Expression of selected genes involved in OXPHOS and anaerobic glycolysis in pluripotent lineages. M: morula (n = 47 biologically independent cells), EB: early blastocyst (n = 24 biologically independent cells), LB: late blastocyst (n = 25 cells), Sph: spherical embryo (n = 48 cells). Boxes show 25-75 percentile values and white line shows median expression. $P<0.05$ determined by Wilcoxon test. M: morula, EB: early blastocyst, LB: late blastocyst, Sph: spherical embryo, EPI: epiblast, ICM: inner cell mass.

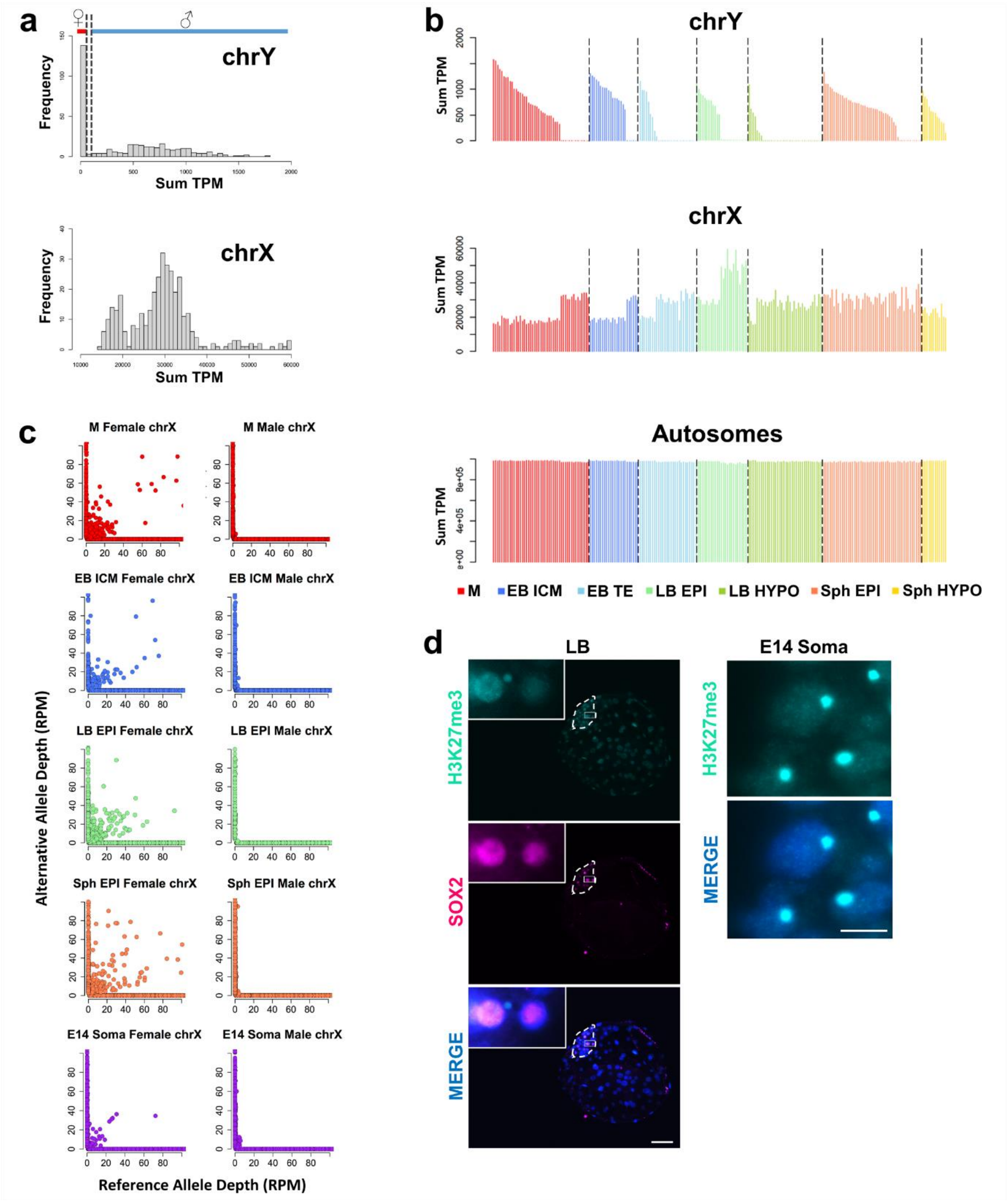

Supplementary Figure 4. Sex determination of pig preimplantation embryos and allelic X chromosome gene expression analysis. a, Histograms showing Y and X chromosomes TPM sum per cell on the x axis and cell frequency on the y axis (see methods for details). **b,** Bar plots of chromosomal TPM sums for sex-classified cells ordered by stages and lineages. **c,** Scatterplots showing allelic expression levels (RPM aligned to the reference and alternative

allele) of all stages and lineages. SNVs with monoallelic expression lie along the axes. **d,** IF staining of H3K27me3, SOX2 and merge with DAPI in a female late blastocyst (LB) (scale bar: 50 µm) and H3K27me3 merged with DAPI in E14 somatic (soma) cells (scale bar: 10 µm). E: embryonic day, M: morula, EB: early blastocyst, LB: late blastocyst, Sph: spherical embryo, EPI: epiblast, HYPO: hypoblast, ICM: inner cell mass, TE: trophectoderm.
