# Supplementary material for: Pluripotency and X chromosome dynamics revealed in pig pre-gastrulating embryos by single cell analysis
Source: Nat Commun. 2019 Jan 30;10:500. doi: 10.1038/s41467-019-08387-8 (PMC6353908; doi:10.1038/s41467-019-08387-8)
Supplement: Supplementary file 3 — Reporting Summary [file 41467_2019_8387_MOESM3_ESM.pdf]

Editorial Note: Parts of this peer review file have been redacted as indicated to remove third-party material where no permission to publish could be obtained.

Reviewers' comments:

Reviewer #1 (Remarks to the Author):

This manuscript provides a comprehensive and very thorough analysis of early lineage segregation, pluripotency status and X-chromosome inactivation in pre-gastrulation pig embryo. To my knowledge, this is novel for the pig. The dataset is very important given the growing role of the pig as a model organism for biomedical research and – in particular – the potential of pig embryos as hosts for human stem cells aiming at the generation of human tissues and organs in pigs by blastocyst complementation. Although several studies on the latter topic have been published in prominent Journals, the efficacy of human stem cells to contribute to the developing chimeric embryos was so far disappointingly low. The dataset provided in the present manuscript may provide new clues how to improve porcine embryos as a niche for the development and differentiation of human stem cells. Detailed insight into the timing and mechanisms of early lineage specification events – as provided by this manuscript – will also strengthen the role of the pig as a model for human development. Recent studies, e.g. on the role of POU5F1/OCT4 in preimplantation embryo development, revealed that mouse embryos do not reflect early human development whereas porcine and bovine embryos are much more similar.
The manuscript is generally very well written and the data well presented. I have a few minor comments.
Abstract & page 3 line 47: The statement that early human post-implantation embryos are inaccessible should be tuned down and the paper “Shahbazi MN, et al. 2016. Self-organization of the human embryo in the absence of maternal tissues. Nat. Cell Biol. 18, 700–708” cited.
Page 3 line 65: please rephrase sentence
Page 5 line 111: It would be interesting to have trophectoderm data also from the advanced stages (LB and Sph embryos). It is clear that TE cells from these stages are difficult to disaggregate, but a piece of TE is easy to separate and could have been analyzed.
Fig. 1c better use “hypoblast” instead of “primitive endoderm”

Reviewer #2 (Remarks to the Author):

The manuscript of Ramos-Ibeas and colleagues aims to provide a better understanding of the lineage segregation processes during preimplantation mammalian development. Specifically, it concentrates on the analysis of dynamic changes in gene expression, metabolism and activity of different signaling pathways during pre and peri-implantation periods of pig development. The authors characterize the gene expression profiles of individual cells form morula stage till spherical embryo stage.
They present a systematic analysis of the progressive segregation of the inner cell mass and trophectoderm in early blastocysts followed by separation of epiblast and hypoblast in late blastocysts. In addition, Ramos-Ibeas and and colleagues postulate that they were able to determine when the transmission form naïve to primed pluripotency takes place in pig embryos.

The manuscript is very timely; it addresses one of the key problems in stem cells research and significantly advances our knowledge of early mammalian development. The data on the lineage segregation in pigs are of potentially high interest for the scientific community working on mammalian development, stem cells derivation and the origin of pluripotency. At the same time, they provide extremely valuable resources for possible further research.
The majority of experiments are carefully planned and conducted, although some of them do not address the issues they were – in authors opinion - designed to address (see details below). Therefore, although I strongly believe that this beautiful piece of work deserve publishing, I urge the authors to

address few important problems.

Major issues:

1. Although this work provides an impressive amount of data and helps to understand the basic principles that drive mammalian development I think the authors overplayed the part about naïve pluripotency. First, I do not understand on what basis the authors call moulae and early blastocyst cells "pluripotent"? According to the most commonly used definition, pluripotency is a feature of cells that have the ability to contribute to all tissues in the adult organism (including germ line) but not to extra-embryonic tissues. From the data presented here, one can assume that moulae and early blastocyst cells give rise to both EPI (embryonic lineage) and HYPO (extraembryonic lineage), unlike the pluripotent cells (as defined from mouse studies) that can be found in EPI only. Moreover, co-expression of pluripotency factors together with HYPO markers at morula and early blastocyst stages in pigs further suggest that naïve pluripotency is not yet established at these stages. Potentially, naïve pluripotency is established at mid blastocyst stage in pigs, however, regretfully, the authors did not provide any single cell data from this stage. Therefore, the authors should both provide additional data and prove that they pinpointed the emergence of naïve pluripotency in pigs, or the wording of the manuscript should be appropriately and carefully changed to more precisely represent their findings.
2. On the same note, it is not clear why the effect of the inhibitor treatment experiments was tested at mid-blastocyst stage (clearly seeing the importance of this stage) but the authors decided not to perform a single cell analysis from this stage.
3. The rationale behind choosing the embryonic stages for inhibitor treatment as well as the length of the treatment itself is not presented very clearly. Why in some experiments embryos were treated from morula stage and in others from early blastocyst stage? Why were not all inhibitor experiments performed in the same way? I assume that this has something to do with the presence or absence of the various components of particular pathways as shown in single cell data but this needs to be clarified, and the regime of treatment needs to be clearly presented for each inhibitor (preferably graphically in the figure)

Minor points:

1. Introduction: line 43. Why do the authors define “early development” as timing between E3.5 and E5.5? How would they call a development period from zygote till blastocyst stage?

2. Figure 2a, d, e, f, g and h are presented in a very confusing way. Why does BF not match fluorescence images? If these are not the same embryos, why show BF at all?

3. Results: line 103 and 104, sentence commencing at: “Notably we found (...). It is not clear what stage the authors are talking about, EB? In fact this is the problem for the whole section of the results. I would strongly advice to revise the this part of the Results and make sure that it is very clear to what stage the authors refer to and what species they talking about when they compare their findings to the published data.

4. Line 137-138, I am assuming that the authors wanted to say “the role of specific signaling pathways during pig development?”

5. Line 153-154, sentence: “The total nr of cells was also reduced, suggesting a role of this pathway in TE development.” Not sure whether this conclusion is fully justified as the authors already mentioned the decrease in number of Nanog positive cells which, in theory, could account for the decrease in the total cell number.

6. Line 226-7, sentence: “Some of these cells also showed SOX17 co-expression” Where are these data visualized? Any numbers? Or these are the data “not showed”?

Reviewer #3 (Remarks to the Author):

In this manuscript entitled “Lineage segregation, pluripotency and X-chromosome inactivation in the pig pre-gastrulation embryo”, the authors performed single cell RNA-seq analysis to investigate the process of early porcine embryonic development. They showed lineage segregation of porcine early blastocysts at the transcriptomic level. Furthermore, they identified signaling pathways that are important for early porcine embryonic development such as JAK/STAT and PI3K/AKT signaling pathways. In addition, they also showed the transition from naïve pluripotency to primed pluripotency during pig early embryonic development, and distinct metabolic programs during the transition of naïve and primed pluripotent states. Finally, they observed dosage compensation of X chromosome during pig embryo development. In general, this is an interesting study that provides molecular insights into pig early embryonic development. However, additional evidence is needed to strengthen their major conclusions in this study.

Specific comments:

1. The authors identified signaling pathways enriched in pig early embryonic development by bioinformatic analysis. These signaling pathways include JAK-STAT, PI3K-AKT, MAPK, and TGFbeta. However, in Supplementary Figure 2b, it is observed that genes regulating PPAR signaling pathway is also enriched. Have the authors analyzed whether PPAR signaling pathway plays a role in pig early embryonic development?

2. In Figure 2, the authors disturbed signaling pathways using small molecules to analyze the influence of signaling pathways (JAK-STAT, PI3K-AKT, TGFbeta, MAPK, and WNT) on pig early embryonic development. It would be important to use cytokines or chemical agonists to activate these signaling pathways during pig early embryonic development and analyze whether activating these signaling pathways would have an effect on pig embryo development. This could provide further evidence to support the importance of these signaling pathways during pig embryo development.

3. In Supplementary Figure 3C, the authors showed that LIF is not expressed during pig early embryo development, whereas IL6 and IL6R are expressed. Have the authors attempted to use anti-IL6 antibody to block IL6 signaling during pig early embryo development? This experiment would be important to clarify whether IL6 is responsible for active JAK-STAT signaling during pig early embryo development.

4. In Figure 4a and 4b, the authors analyzed the expression of genes involved in oxidative phosphorylation and glycolysis, and found a metabolic shift from oxidative phosphorylation to glycolysis during the transition from naïve pluripotency to primed pluripotency. This conclusion would be strengthened if additional evidence in addition to gene expression is provided. For example, have the authors analyzed the level of L-lactate in the culture suspension during the culture of pig embryos?

5. In Figure 4c, the authors analyzed the mRNA expression of important epigenetic regulators during pig early embryonic development, and suggested that there is a gradual increase of DNA methylation during this process. Have the authors analyzed the level of DNA methylation during pig early embryonic development?

Point-by-point reply to the reviewers' comments:

We thank the reviewers for their evaluation of our manuscript and recommendations made. We believe that the manuscript has been improved with the addition of new experimental data addressing the reviewer's suggestions. The changes to the manuscript are highlighted in yellow in the text. We have also changed Figure 2, and added information to Supplementary Fig. 4b to address the reviewer's comments. Below is a detailed response to reviewers' comments in *blue italics*:

Reviewer #1 (Remarks to the Author): This manuscript provides a comprehensive and very thorough analysis of early lineage segregation, pluripotency status and X-chromosome inactivation in pre-gastrulation pig embryo. To my knowledge, this is novel for the pig. The dataset is very important given the growing role of the pig as a model organism for biomedical research and – in particular – the potential of pig embryos as hosts for human stem cells aiming at the generation of human tissues and organs in pigs by blastocyst complementation. Although several studies on the latter topic have been published in prominent Journals, the efficacy of human stem cells to contribute to the developing chimeric embryos was so far disappointingly low. The dataset provided in the present manuscript may provide new clues how to improve porcine embryos as a niche for the development and differentiation of human stem cells. Detailed insight into the timing and mechanisms of early lineage specification events – as provided by this manuscript – will also strengthen the role of the pig as a model for human development. Recent studies, e.g. on the role of POU5F1/OCT4 in preimplantation embryo development, revealed that mouse embryos do not reflect early human development whereas porcine and bovine embryos are much more similar.
The manuscript is generally very well written and the data well presented. I have a few minor comments.

Abstract & page 3 line 47: The statement that early human post-implantation embryos are inaccessible should be tuned down and the paper "Shahbazi MN, et al. 2016. Self-organization of the human embryo in the absence of maternal tissues. Nat. Cell Biol. 18, 700–708" cited.

We have rephrased the abstract and added the reference to Shahbazi et al., (2016) as suggested, as well as citation of a similar study by Deglincerti et al., (2016). (Line 29 and 49).

Page 3 line 65: please rephrase sentence

This has been rewritten (Line 60-64).

Page 5 line 111: It would be interesting to have trophectoderm data also from the advanced stages (LB and Sph embryos). It is clear that TE cells from these stages are difficult to disaggregate, but a piece of TE is easy to separate and could have been analyzed.

The process of TE differentiation during pig development was described in great detail previously (Blomberg et al., 2005, Phys Genomics 20:1880194; Blomberg et al., 2010 Mol Rep Dev 77:978-989), which was not therefore the focus of our current research. The focus of our study was to gain insight of the less well-understood question of lineage segregation in developing E6 blastocysts. Our findings however will inform new studies to gain further understanding of TE differentiation in developing pig embryos.

Fig. 1c better use “hypoblast” instead of “primitive endoderm”

This has been changed as suggested.

Reviewer #2 (Remarks to the Author):

The manuscript of Ramos-Ibeas and colleagues aims to provide a better understanding of the lineage segregation processes during preimplantation mammalian development. Specifically, it concentrates on the analysis of dynamic changes in gene expression, metabolism and activity of different signaling pathways during pre and peri-implantation periods of pig development. The authors characterize the gene expression profiles of individual cells form morula stage till spherical embryo stage. They present a systematic analysis of the progressive segregation of the inner cell mass and trophectoderm in early blastocysts followed by separation of epiblast and hypoblast in late blastocysts. In addition, Ramos-Ibeas and colleagues postulate that they were able to determine when the transmission form naïve to primed pluripotency takes place in pig embryos.

The manuscript is very timely; it addresses one of the key problems in stem cells research and significantly advances our knowledge of early mammalian development. The data on the lineage segregation in pigs are of potentially high interest for the scientific community working on mammalian development, stem cells derivation and the origin of pluripotency. At the same time, they provide extremely valuable resources for possible further research. The majority of experiments are carefully planned and conducted, although some of them do not address the issues they were – in authors opinion - designed to address (see details below). Therefore, although I strongly believe that this beautiful piece of work deserve publishing, I urge the authors to address few important problems.

Major issues:

1. Although this work provides an impressive amount of data and helps to understand the basic principles that drive mammalian development I think the authors overplayed the part about naïve pluripotency. First, I do not understand on what basis the authors call morulae and early blastocyst cells "pluripotent"? According to the most commonly used definition, pluripotency is a feature of cells that have the ability to contribute to all tissues in the adult organism (including germ line) but not to extra-embryonic tissues. From the data presented here, one can assume that moulae and early blastocyst cells give rise to both EPI (embryonic lineage) and HYPO (extraembryonic lineage), unlike the pluripotent cells (as defined from mouse studies) that can be found in EPI only. Moreover, co-expression of pluripotency factors together with HYPO markers at morula and early blastocyst stages in pigs further suggest that naïve pluripotency is not yet established at these stages. Potentially, naïve pluripotency is established at mid blastocyst stage in pigs, however, regretfully, the authors did not provide any single cell data from this stage. Therefore, the authors should both provide additional data and prove that they pinpointed the emergence of

naïve pluripotency in pigs, or the wording of the manuscript should be appropriately and carefully changed to more precisely represent their findings.

The scRNAseq sample collection was designed to obtain representative stages over a 6-7 day period (from E4/5-E10/11). We reasoned that collecting samples at distinct stages would provide a clear gene expression profile and unambiguous separation of cellular trajectories. For our early stages (E4/5, E5/6, and E7/8) a sampling interval of less than one day was used, which considering the asynchronous nature of ovulation (between 12-24 hrs) in pigs, it is the best we could do in terms of collecting in vivo produced embryos at short intervals.

The PCA analysis of M/ICM cells (E4-6) shows that these cells express many genes found in human and mouse naïve pluripotent cells, but they also show expression of HYPO genes, as indicated by the reviewer. Cells collected from one day older embryos (E7-8) express genes typically found in primed pluripotent cells, with no overlapping HYPO gene expression (Fig. 1c; SF2c,d,e). These findings are consistent with observations in mouse (Mohammed et al., 2016 Cell Reports 20:1215-28) and monkey (Nakamura et al., 2015 531:57-62) where naïve pluripotent gene expression was described in the early ICM. This was used to describe transitional properties of pluripotent cells, although HYPO genes (PDGFRα, GATA6) were still expressed in these ICM cells. Following the reviewers suggestion we have now reworded the description of our findings to indicate that <u>gene expression profiles</u> consistent with a naïve or a primed profile could be identified in our dataset, rather than suggesting cellular states.

Based on our sampling interval of no more than 1 day between EB and LB we believe to have captured progressive representative stages of the developing pig embryo. Based on our findings, our working hypothesis for the emergence of pluripotency in the pig embryo is that ICM cells first express markers of Naïve pluripotency (KLF4/5/17, TBX3) concurrently with early HYPO markers (GATA6/PDGFRα), and these cells gradually (within ~1 day) will transition to a gene expression profile associated with primed EPI (NODAL, ZIC3, DNMT3B) or HYPO (GATA4, COL4A1, NID2), respectively. Morula cells, which also express naïve markers, do not express NANOG, but they also express the TE markers GATA2/3 and DAB2, indicating that that these cells also have the potential to form TE.

We have reworded this section of the paper to reflect the points raised by the referee and discussed above.

2. On the same note, it is not clear why the effect of the inhibitor treatment experiments was tested at mid-blastocyst stage (clearly seeing the importance of this stage) but the authors decided not to perform a single cell analysis from this stage.

The results in figure 2 are based on a classification done on embryos that are monitored closely in the laboratory from the morula stage, and where early, mid and late blastocysts can be precisely sampled during in vitro culture. Such close monitoring cannot be done from in vivo derived embryos. Based on the findings from scRNAseq we used the in vitro culture system to gain detailed understanding of the signalling pathways operating during the window of development presented (early, mid and late Blastocyst). Treatment to the mid-blastocyst stage was done for all treatments. For TGFB1i we extended the cultures to LB, to match the findings of the RNASeq showing upregulation of this signalling pathway in LB embryos. Indeed, in LB embryos the number of NANOG cells was reduced when TGFB1 was blocked. In the case of Jaki inhibition however the effect was observed from EB stage, consistent with the expression of Jak signalling component detected in early embryos.

3. The rationale behind choosing the embryonic stages for inhibitor treatment as well as the

length of the treatment itself is not presented very clearly. Why in some experiments embryos were treated from morula stage and in others from early blastocyst stage? Why were not all inhibitor experiments performed in the same way? I assume that this has something to do with the presence or absence of the various components of particular pathways as shown in single cell data but this needs to be clarified, and the regime of treatment needs to be clearly presented for each inhibitor (preferably graphically in the figure).

The embryonic stages used for the different treatments were selected based on the expression of each of the specific signalling pathways under investigation, as explained above. This is indicated in the text (L.142). We have reorganized figure 2 and include a clearer outline of the treatments and a rationale for each treatment in the text.

Minor points:

1. Introduction: line 43. Why do the authors define “early development” as timing between E3.5 and E5.5? How would they call a development period from zygote till blastocyst stage?

We have re-worded the sentence to reflect early development as the whole period until the formation of the epiblast.

2. Figure 2a, d, e, f, g and h are presented in a very confusing way. Why does BF not match fluorescence images? If these are not the same embryos, why show BF at all?

We have completely reorganized this figure, leaving the representative IF images 2a, and presenting the inhibitor experiments results in the scatter-plots.

3. Results: line 103 and 104, sentence commencing at: “Notably we found (...). It is not clear what stage the authors are talking about, EB? In fact this is the problem for the whole section of the results. I would strongly advice to revise the this part of the Results and make sure that it is very clear to what stage the authors refer to and what species they talking about when they compare their findings to the published data.

We are grateful to the reviewer for pointing out that our descriptions are unclear; we apologize for the confusion. We have now revised the results to identify clearly the stages described.

4. Line 137-138, I am assuming that the authors wanted to say “the role of specific signaling pathways during pig development?”

Thank you for pointing this out. We have indicated during pig development, as suggested.

5. Line 153-154, sentence: “The total nr of cells was also reduced, suggesting a role of this pathway in TE development.” Not sure whether this conclusion is fully justified as the authors already mentioned the decrease in number of Nanog positive cells which, in theory, could account for the decrease in the total cell number.

We accept the reviewer’s comment and address it in the revised manuscript. The reduction in NANOG cells is significant, from an average of 10-12 to ~3 NANOG positive cells per embryo in the treated group (a reduction of about 10 cells/embryo). However the total cell

number was reduced by more than 35 cells/embryo, indicating that the number of TE cells was also reduced. We explain this finding more fully in the text (Line 168).

6. Line 226-7, sentence: “Some of these cells also showed SOX17 co-expression” Where are these data visualized? Any numbers? Or these are the data “not showed”?

The data is now added in Supplementary Figure 4 and is described in this section of the results (Line 237).

Reviewer #3 (Remarks to the Author):

In this manuscript entitled “Lineage segregation, pluripotency and X-chromosome inactivation in the pig pre-gastrulation embryo”, the authors performed single cell RNA-seq analysis to investigate the process of early porcine embryonic development. They showed lineage segregation of porcine early blastocysts at the transcriptomic level. Furthermore, they identified signaling pathways that are important for early porcine embryonic development such as JAK/STAT and PI3K/AKT signaling pathways. In addition, they also showed the transition from naïve pluripotency to primed pluripotency during pig early embryonic development, and distinct metabolic programs during the transition of naïve and primed pluripotent states. Finally, they observed dosage compensation of X chromosome during pig embryo development. In general, this is an interesting study that provides molecular insights into pig early embryonic development. However, additional evidence is needed to strengthen their major conclusions in this study.

Specific comments:

1. The authors identified signaling pathways enriched in pig early embryonic development by bioinformatic analysis. These signaling pathways include JAK-STAT, PI3K-AKT, MAPK, and TGFbeta. However, in Supplementary Figure 2b, it is observed that genes regulating PPAR signaling pathway is also enriched. Have the authors analyzed whether PPAR signaling pathway plays a role in pig early embryonic development?

Our focus was on JAK-STAT, PI3K-AKT, MAPK, and TGFbeta because of the known roles of these signalling pathways in early embryo development in different species, and here they were studied in detail in the pig. In Suppl. Fig 2 we also present genes of other pathways expressed at different stages and may be operating during pig development, including PPAR signalling. PPAR shows significant upregulation in Hypoblast and Epiblast of spherical embryos, and this expression is consistent with previous publications showing high expression in Day 11 pig conceptuses, suggesting that this pathway may participate in trophoblast elongation Blitek et al., (2017, 101:53-61).
However, our aim was to gain a better understanding of lineage segregation and pluripotency establishment, therefore we did not consider it a priority to investigate PPAR signalling in detail in our study. We expect that readers of the manuscript will find the information provided useful and that future experiments will investigate in more detail the novel pathways described in our report.

2. In Figure 2, the authors disturbed signaling pathways using small molecules to analyze the influence of signaling pathways (JAK-STAT, PI3K-AKT, TGFbeta, MAPK, and WNT) on pig early embryonic development. It would be important to use cytokines or chemical agonists to activate these signaling pathways during pig early embryonic development and

analyze whether activating these signaling pathways would have an effect on pig embryo development. This could provide further evidence to support the importance of these signaling pathways during pig embryo development.

We agree with this suggestion and previous studies from our lab and others have shown effects of some of the cytokines referred to by the reviewer: 1) we previously showed that stimulation of MAPK by FGF completely eliminates NANOG positive cells in pig blastocysts, indicating that MAPK signalling promotes hypoblast segregation (Rodriguez et al., 2012; PLoS One 7(11):e49079. 2) Stimulation of WNT using GSK3bi reduces pig blastocyst development, suggesting a role for WNT during TE development (Huang et al., 2013 Cellular Sig 25:778-785; Lim et al., 2013, Theriogenology 79:284-90).
The PI3K/Akt signalling pathways transduces signals from many ligands (EGF, Insulin, IGF-1, FGF, shh) and therefore it is not possible to stimulate this signalling specifically.
In response to the reviewer's suggestion we assessed the effect of TGFB1 on blastocyst development and lineage segregation and the results are presented in L170.
We also stimulated the JAK-Stat signalling with IL-6 supplementation, based on the high expression detected in our dataset. Furthermore, an earlier study reported positive effects of IL-6 in pig parthenogenetic embryos (Shen et al., 2012 J Reprod Dev, 58:453-60). Our results are now presented in L149 and discussed in L332-349.

3. In Supplementary Figure 3C, the authors showed that LIF is not expressed during pig early embryo development, whereas IL6 and IL6R are expressed. Have the authors attempted to use anti-IL6 antibody to block IL6 signaling during pig early embryo development? This experiment would be important to clarify whether IL6 is responsible for active JAK-STAT signaling during pig early embryo development.

This is a valuable suggestion and we have investigated the effect of IL-6 modulation during pig embryo development by inactivating IL-6 expression in CRISPR/Cas9 gene edited embryos (see Fig. 2f). This information has now added further evidence of the important role of IL-6 in TE development, supporting the findings using the Jak-STAT specific inhibitor.

4. In Figure 4a and 4b, the authors analyzed the expression of genes involved in oxidative phosphorylation and glycolysis, and found a metabolic shift from oxidative phosphorylation to glycolysis during the transition from naïve pluripotency to primed pluripotency. This conclusion would be strengthened if additional evidence in addition to gene expression is provided. For example, have the authors analyzed the level of L-lactate in the culture suspension during the culture of pig embryos?

This is a good point and we should have referred to a previous study that measured L-Lactate production during pig embryo development. We have now cited this report (L383), which in agreement with our findings, shows that during the morula to blastocyst transition pig embryos shift from aerobic respiration to anaerobic glycolysis resulting in high production of Lactate as development progresses and a concomitant reduction in ATP levels and oxygen consumption (Sturmey et al., 2003 Reproduction 126:197-204). This demonstrates that pig embryos, like other mammals, reduce aerobic respiration during late stages of blastocyst development.

Redacted

Figure taken from *Sturmey et al.,* (2003) Reproduction 126:197-204

5. In Figure 4c, the authors analyzed the mRNA expression of important epigenetic regulators during pig early embryonic development, and suggested that there is a gradual increase of DNA methylation during this process. Have the authors analyzed the level of DNA methylation during pig early embryonic development?

DNA methylation levels in pig embryos have previously been reported by other studies. They show that, like in other mammals, 5mC and 5hmC are present in the porcine ICM (Cao et al., 2014, Theriogenology 81:496-508; Lee et al., 2014 Dev Biol 386:86-95). Genome wide analysis of DNA methylation of blastocysts shows a low level of methylation (13%) compared to somatic tissues (~75%) (Canovas et al., 2017 eLIFE 6:e23760). This is consistent with our observation that blastocysts express low levels of DNMT3A/B, however during the maturation of the epiblast in late blastocysts and spherical embryos these enzymes become highly expressed, suggesting a gradual increase of methylation during development.

REVIEWERS' COMMENTS:

Reviewer #1 (Remarks to the Author):

Thank you for addressing my comments in the revised Version of the manuscript.

Reviewer #2 (Remarks to the Author):

In the revised version of the manuscript, Ramos-Ibeas and colleagues sufficiently addressed all my main concerns and I am satisfied with their response to my comments. It is my pleasure to recommend this manuscript for publication in Nature Communications.

There is only one minor issue that needs additional attention. Namely, in the discussion section, line 356; sentence needs revision: “Analysis of embryonic cells revealed an expression profile consistent with naïve pluripotency “– Is is still an overstatement. The expression profile was consistent with undetermined ICM state – as observed in very early mouse ICMs where pluripotency factors are expressed together with Hypoblast specific factors. Naïve pluripotency, as defined in mouse, is characterized by exclusive expression of pluripotency factors with Hypoblast factors being downregulated.
I am guessing the authors wanted to say is that factors characteristic for naïve pluripotency were present in pig embryos at M and EB stages.

Reviewer #3 (Remarks to the Author):

I have read through the revised manuscript and the responses from the authors. My concerns have all been solved and I have no further questions.

<u>Point-by-point reply to the reviewers' comments</u>:

Below is a response (*in blue italics)* to the reviewers' comments:

REVIEWERS' COMMENTS:

Reviewer #1 (Remarks to the Author):

Thank you for addressing my comments in the revised Version of the manuscript.

No issues raised.

Reviewer #2 (Remarks to the Author):

In the revised version of the manuscript, Ramos-Ibeas and colleagues sufficiently addressed all my main concerns and I am satisfied with their response to my comments. It is my pleasure to recommend this manuscript for publication in Nature Communications.

There is only one minor issue that needs additional attention. Namely, in the discussion section, line 356; sentence needs revision: "Analysis of embryonic cells revealed an expression profile consistent with naïve pluripotency "– Is is still an overstatement. The expression profile was consistent with undetermined ICM state – as observed in very early mouse ICMs where pluripotency factors are expressed together with Hypoblast specific factors. Naïve pluripotency, as defined in mouse, is characterized by exclusive expression of pluripotency factors with Hypoblast factors being downregulated.
I am guessing the authors wanted to say is that factors characteristic for naïve pluripotency were present in pig embryos at M and EB stages.

We thank the reviewer for the thoughtful suggestion. We have now changed the sentence in L358 as suggested by the reviewer: "Analysis of embryonic cells revealed an expression profile with genes characteristic of naïve pluripotency in morula and ICM cells".

Reviewer #3 (Remarks to the Author):

I have read through the revised manuscript and the responses from the authors. My concerns have all been solved and I have no further questions.

No issues raised.
